# Correcting for base-population differences and unknown parent groups in single-step genomic predictions of Norwegian Red cattle

**DOI:** 10.1093/jas/skac227

**Published:** 2022-06-25

**Authors:** Tesfaye K Belay, Leiv S Eikje, Arne B Gjuvsland, Øyvind Nordbø, Thierry Tribout, Theo Meuwissen

**Affiliations:** Department of Animal and Aquacultural Sciences, Norwegian University of Life Sciences, 1432 Ås, Norway; GENO SA, Storhamargata 44, 2317 Hamar, Norway; GENO SA, Storhamargata 44, 2317 Hamar, Norway; GENO SA, Storhamargata 44, 2317 Hamar, Norway; Université Paris Saclay, INRAE, AgroParisTech, GABI, 78350 Jouy-en-Josas, France; Department of Animal and Aquacultural Sciences, Norwegian University of Life Sciences, 1432 Ås, Norway

**Keywords:** genetic groups, inflation, J factor, level-bias, Norwegian Red cattle, single-step genomic BLUP

## Abstract

Bias and inflation in genomic evaluation with the single-step methods have been reported in several studies. Incompatibility between the base-populations of the pedigree-based and the genomic relationship matrix (**G**) could be a reason for these biases. Inappropriate ways of accounting for missing parents could be another reason for biases in genetic evaluations with or without genomic information. To handle these problems, we fitted and evaluated a fixed covariate (**J**) that contains ones for genotyped animals and zeros for unrelated non-genotyped animals, or pedigree-based regression coefficients for related non-genotyped animals. We also evaluated alternative ways of fitting the **J** covariate together with genetic groups on biases and stability of breeding value estimates, and of including it into **G** as a random effect. In a whole vs. partial data set comparison, four scenarios were investigated for the partial data: genotypes missing, phenotypes missing, both genotypes and phenotypes missing, and pedigree missing. Fitting **J** either as fixed or random reduced level-bias and inflation and increased stability of genomic predictions as compared to the basic model where neither **J** nor genetic groups were fitted. In most models, genomic predictions were largely biased for scenarios with missing genotype and phenotype information. The biases were reduced for models which combined group and **J** effects. Models with these corrected group covariates performed better than the recently published model where genetic groups were encapsulated and fitted as random via the Quaas and Pollak transformation. In our Norwegian Red cattle data, a model which combined group and **J** regression coefficients was preferred because it showed least bias and highest stability of genomic predictions across the scenarios.

## Teaser

Towards an unbiased and stable combination of information from genotyped and non-genotyped animals in genomic prediction models.

## Introduction

Unbiased predictions of breeding values are crucial in selections across heterogeneous groups of selection candidates (e.g., different ages or genotyped and non-genotyped) and for accurate estimation of genetic trend. Genomic predictions with the single-step genomic BLUP (SSGBLUP) approach are expected to yield unbiased genomic estimated breeding value (GEBV) as it combines all available data from genotyped and non-genotyped animals in one analysis (Legarra et al., 2009; Aguilar et al., 2010; Christensen and Lund, 2010). This integration should allow information on unselected animals to be included, with all relationships tracing back to a conceptual unselected base-population. The SSGBLUP method assumes that genomic relationships (**G**) and pedigree relationship (**A**) matrices refer to the same base-population. However, this assumption may not hold in practice as **A** and **G** refer to different base-populations. Incompatibility between the base-populations of the **A** and **G** matrices could be one of the reasons for biases observed in SSGBLUP genomic predictions. To handle this problem, VanRaden (2008) suggested the use of base-population allele frequencies when computing **G** to achieve compatibility of **G** and **A**. However, base-population allele frequencies are rarely available in practice since base animals are not genotyped (Powell et al., 2010; Christensen, 2012) though such frequencies can be estimated as in Gengler et al. (2007) and Aldridge et al. (2019).

Several studies have discussed this problem and proposed solutions for the SSGBLUP procedure (Meuwissen et al., 2011; Vitezica et al., 2011; Christensen, 2012; Legarra et al., 2015) or for the ssSNPBLUP model (Fernando et al., 2014; Fernando et al., 2016; Hsu et al., 2017). Fernando et al. (2014) proposed to fit a fixed covariate (**J**) that contains ones for genotyped animals and zeros for non-genotyped animals, whose genotypes could also not be imputed (i.e., those that are not related to genotyped animals), and otherwise sums of the regression coefficients used for genotype imputation. Hsu et al. (2017) fitted the **J** factor in the ssSNPBLUP model using simulated data and observed an increased accuracy when **J** was included in the model for populations under selection. They showed that estimating the effect of this covariate $\left(\mu_{g}\right)$ implicitly estimates the (base) allele frequency by which the marker genotype codes should be centered. The latter is thus estimated from the data by estimating this intercept. The covariate $J\mu_{g}$ of Fernando et al. (2014) and Hsu et al. (2017) is very similar to the covariate α in Vitezica et al. (2011) and Chen et al. (2011). The difference is that in the first case it is fitted as fixed and explicitly estimated, and in the second case it is random and absorbed into **G**.

In a simulation study, Bermann et al. (2021) extended the **J** covariate from Hsu et al. (2017) to the SSGBLUP approach and fitted it as a fixed effect. Vandenplas et al. (2021) fitted the **J** covariate as a fixed effect for milk and temperament in Dutch and Belgium dairy cattle using the ssSNPBLUP model. The **J** covariate could be fitted as a fixed variable in the SSGBLUP model (Bermann et al., 2021) and would account for a possible genetic difference between the non-genotyped and genotyped animals (Vitezica et al., 2011; Fernando et al., 2014; Hsu et al., 2017; Bermann et al., 2021). However, it does not correct for any differences in the variance of genetic relationships that may result due to differences in base-populations, which was addressed by Legarra et al. (2015). The **J** covariate also accounts for part of the genetic difference between genotyped and non-genotyped animals that can be explained by genotype imputation. Due to the latter, the **J** covariate can affect biases and accuracy of prediction (Fernando et al., 2014; Hsu et al., 2017). Thus, the **J** factor correction may improve genomic predictions of the ssSNPBLUP (Hsu et al., 2017) and the SSGBLUP (Vitezica et al., 2011; Bermann et al., 2021) models using simulated data, and we want to confirm and quantify these improvements here in Norwegian Red cattle data using SSGBLUP.

Another reason for bias and inflation in single-step genomic predictions (and in pedigree-based BLUP) could be due to inappropriate corrections for missing parents (genetic groups) that could come from several founder populations. The genetic groups account for genetic differences among those founder populations and ignoring them in genetic evaluations would result in biased predictions (Kennedy, 1981; Quaas, 1988). Several studies modeled genetic groups in single-step genomic evaluations context (Misztal et al., 2013; Bradford et al., 2019; Tsuruta et al., 2019) using different strategies that have recently been summarized by Masuda et al. (2021) who also proposed a new strategy for modeling group effects. Effects of genetic groups on bias and accuracy in those studies varied based on sources of the genetic groups (either from **A** or combined relationship matrix **H**), amount of information available in defining genetic groups, strategies used to model them and trait heritability. Genetic groups can also be fitted together with the **J** factor in genomic evaluations (Tsuruta et al., 2019; Bermann et al., 2021; Vandenplas et al., 2021). Alternative ways of combining the genetic groups with the **J** factor were envisaged and evaluated here.

Hence, the objective of this study was to evaluate alternative approaches to fitting genetic groups and **J** factor on biases and a parameter related to accuracy (stability) of SSGBLUP evaluations in Norwegian Red cattle. Our results are thus applicable to breeding value evaluations where both genetic group and **J** factor corrections are required, which is the case in many situations. In addition, we compared fitting the **J** factor and genetic group effects as fixed or as random effects as proposed by Vitezica et al. (2011) and Masuda et al. (2021).

## Materials and Methods

Animal Care and Use Committee approval was not obtained for this study because the data were obtained from an existing database supplied by GENO SA (https://www.geno.no).

### Theory

Fernando et al. (2014) and Hsu et al. (2017) described the theoretical background for deriving and fitting of the **J** covariate that implicitly adjusts for the allele frequencies of the genotype data in the ssSNPBLUP model. As the ssSNPBLUP is equivalent to the SSGBLUP model, this approach towards adjusting of genotypes can be applied to both methods (Hsu et al., 2017). Bermann et al. (2021) derived SSGBLUP equations that are equivalent to the method proposed by Hsu et al. (2017). Our procedure to calculate the **J** factor follows the Fernando et al. (2014) approach and is briefly described below.

Let **M**_**2**_ denote the matrix of genotypes for genotyped individuals and ${\hat{M}}_{1}$ denote the matrix of imputed genotypes for individuals that are not genotyped using **A** matrix-based regression coefficients, i.e., ${\hat{M}}_{1}=A_{12}A_{22}^{-1}M_{2}$ with denoting the block of the $A_{12}$**A** matrix that pertains to non-genotyped (1) and genotyped animals (2), and $A_{22}$ denoting the pedigree relationships between the genotyped animals. The model for the genotypic values of non-genotyped individuals, $g_{1}$ and genotyped individuals, $\;\;\;g_{2}$ is given by Equation 4 of Hsu et al. (2017) as


$$\begin{array}{l} g=1\mu +J\mu_{g}+M\alpha +\epsilon , \end{array}$$

where $g={\left[g_{1}^{\prime }g_{2}^{\prime }\right]}^{\prime }$**M** is a matrix of imputed $\left({\hat{M}}_{1}\right)$ and observed (**M**_**2**_) genotypes; α is a vector of marker genotype effects; ϵ is a vector of imputation residuals for non-genotyped animals: ϵ is the part of the genotypic value that cannot be predicted from imputed marker genotypes (due to imputation inaccuracies) and is predicted using pedigree relationships in SSGBLUP; μ is the overall mean and equals the expected genetic value of non-genotyped animals without pedigree relationships to genotyped animals; $\mu_{g}$ is the intercept of the regression of the marker genotypes, i.e., it is the average genotypic value of an hypothetical animal *i* with genotypes at all markers, $M_{i}$, equal to the mean genotype $\left(E\left(M_{i}\right)\right)$, i.e., $\mu_{g}=\alpha^{\prime }E\left(M_{i}\right)$ (Hsu et al., 2017) and the **J** covariate is

$J=\left[\begin{array}{l} J_{1}\\ J_{2} \end{array}\right]=\left[\begin{array}{l} A_{12}A_{22}^{-1}1\\ 1 \end{array}\right]$ , which can be obtained efficiently, using partitioned inverse results, by solving the easily formed very sparse system, where $A^{11}J_{1}=-A^{12}J_{2}$ yielding $J_{1}=-{\left(A^{11}\right)}^{-1}A^{12}J_{2}$ (Fernando et al., 2014). In case of millions of non-genotyped descendants of genotyped animals (as in dairy cattle), the **J** covariate is readily computed using the method proposed by Tribout et al. (2019). It may be noted that the sign of **J** is switched here relative to Hsu et al. (2017), but this does not affect the regression model.

### Phenotype, genotype, and pedigree data

Phenotypes on first lactation milk yield were provided by GENO SA (https://www.geno.no) from their national routine genetic evaluations on 3,390,184 Norwegian Red cows, with lactation data from 1979 and onwards. Descriptive statistics of lactation milk yield are presented in Table 1. A pedigree containing 4,624,098 animals that linked animals with records (cows) and bulls was also available. Genotype data were also provided by GENO SA on 30,729 animals (cows and bulls), of which 10,989 animals had phenotypic records. The genotype data consisted of 30,300 single nucleotide polymorphisms (SNP) markers on 29 autosomes for Norwegian Red cattle. Prior to 2015, animals were genotyped on Affymetrix 25K and Illumina BovineSNP50K (v1 and v2) and from 2015 genotyping has been done on a customized 50K Affymetrix chip (see (Nordbø et al., 2019) for details). Markers with Mendelian inconsistency across chips were removed and the final SNP set is the overlap of high-quality SNP between the 50K chips. Missing genotypes were imputed with FImpute v2.2 (Sargolzaei et al., 2014). Among genotyped animals, 674 young cows with records were selected for validation. The young cows selected for validation could have progeny, but their progeny did not have phenotypic information to avoid including information from such close relatives. Different scenarios were considered for the validation animals where their phenotypes, or genotypes, or both phenotypes and genotypes, or pedigree information was missing (see “Evaluation of models” section for detailed descriptions of scenarios).

**Table 1.** Number of records (N) and descriptive statistics of **J** covariate and lactation milk yield in tons (T)

Item^1^	*N*	Mean	SD	Min	Max
Milk yield, T	3,390,184	6.58	1.39	0.56	19.70
J_1_	4,593,369	0.79	0.29	−0.01	1.68
J_2_	30,729	1	0	1	1

**J**_**1**_ and **J**_**2**_ are part of the **J** covariate vector that pertain to non-genotyped and genotyped animals in pedigree, respectively.

### The J factor and genetic groups

Values for the **J** factor were derived for millions of animals in the pedigree using the aforementioned formula in the Julia computing environment (Bezanson et al., 2017). New **J** values were also calculated after setting parents of validation animals to missing in the pedigree (for missing pedigree scenario). The **J** factor was also modified as J* = **1-J** (i.e., genotyped animals had zero **J** values) which was used to modify genetic group (**Q)** contributions when it was necessary.

**Figure 1.**
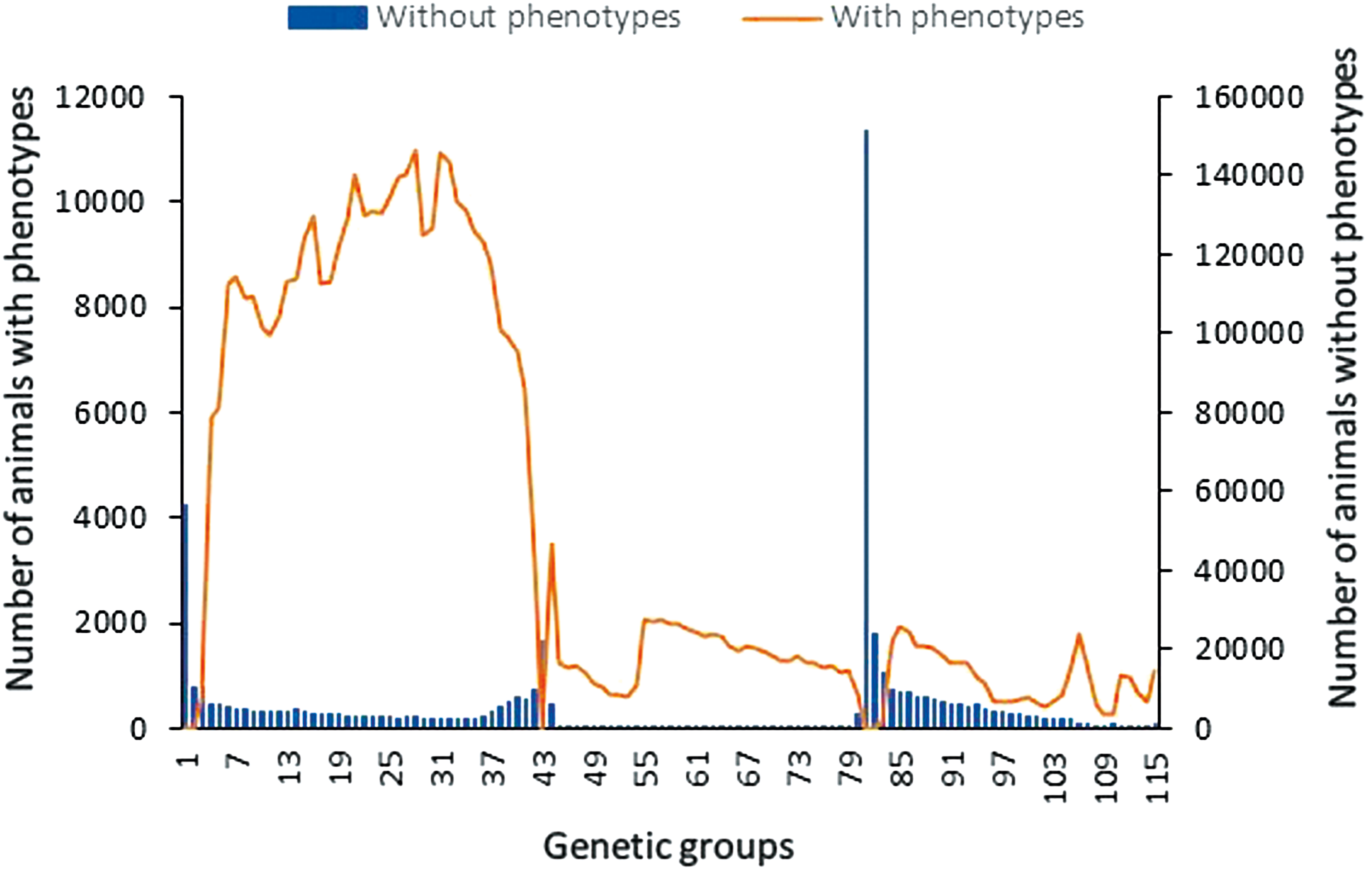
Distribution of animals with or without phenotypes per genetic group.

Missing parents were grouped by year of birth and by the following classes: the missing parent is a missing on-farm bull or a missing AI sire or a missing dam. This resulted in 115 groups which were fitted as fixed covariates or as a random variable following Masuda et al. (2021)’s EUPG (encapsulated unknown parent groups) method. New **Q** contributions were also calculated after setting both parents of animals in the validation group to missing. This aimed to investigate effects of missing pedigree on genomic predictions. Figure 1 presents distribution of animals with or without phenotypes per genetic group. Different **Q** variants or combination of **J** and **Q** (thereof: Q*, **Q**^** + **^and **Q-Q**^**+**^, see below) were created using either the **J** or J* covariate, and the **Q** variants and **J** covariate were illustrated using an example pedigree (see Supplementary Appendix A).

### Combing J and genetic groups

Let us first consider, a situation where the breeding value evaluation consists of two populations A and B, without much missing pedigree. We fit the population effects by a genetic group correction. However, the differences between genotyped and non-genotyped animals may be different in populations A and B. Hence, we would need to fit two **J** factors: one for each population. With more complicated genetic group structures, the fitting of the **J** factor per genetic group becomes more complicated but approaches to this problem are developed below.

Second, consider a single population where all animals are genotyped, and GBLUP is applied. This requires constructing the **G** matrix based on the genotypes, but pedigree is not required. Hence, there can be no missing pedigree, and no genetic group corrections due to missing pedigree are required. This suggests that in SSGBLUP genetic group corrections should be applied to pedigree relationships only and not to **G** (Masuda et al., 2021). The Q* and **Q-Q**^** + **^approaches, described below, attempt to correct non-genotyped animals for genetic group and **J** factor effects whilst avoiding such corrections for genotyped animals. Since the Q* and **Q-Q**^** + **^corrections have 0 coefficients for genotyped animals (see below), the relationships among genotyped animals remain **G** even, e.g., after absorption of the **Q-Q**^** + **^effects or Quaas and Pollak (QP) transformation.

One way to achieve this is to fit a group times J^*^ effect, noting that fitting J^*^ is equivalent to **J** and J^*^ has zero coefficients for genotyped animals. To fit this **Q** times J^*^ effect, we fit a Q* matrix whose columns are obtained by multiplying the columns of **Q** by J^*^ on an element-by-element basis. Since Q* has zero coefficients for the genotyped animals (e.g., Supplementary Appendix Table A1), fitting Q* does not correct genotyped animals for genetic groups.

The **Q**^** + **^matrix was obtained by assuming that the genetic groups also affect the mean of the marker genotypes $E\left(M_{i\left(k\right)}\right),$ and thus, $\mu_{gk}=\alpha^{\prime }E\left(M_{i\left(k\right)}\right)$ for group k. Hence, every group k obtains its own regression coefficient, which $\mu_{gk}$ was estimated following Hsu et al. (2017). For the genotyped animals (2), regression is thus on $Q_{2}^{+}=Q_{2}$, i.e., the usual genetic group contributions (which replaces **J**_**2**_** = 1** in the **J** factor), and for the non-genotyped animals (1), these coefficients are imputed in the same manner as **J**_**1**_, i.e.,. $Q_{1}^{+}=A_{12}A_{22}^{-1}Q_{2}^{+}$. Hence, $Q^{+}=\left[\begin{array}{l} Q_{1}^{+}\\ Q_{2}^{+} \end{array}\right]=\left[\begin{array}{l} A_{12}A_{22}^{-1}Q_{2}\\ Q_{2} \end{array}\right]$, where $Q_{1}^{+}$ and are the blocks of the $Q_{2}^{+}$**Q**^** + **^covariates for non-genotyped and genotyped animals, respectively. The $Q-Q^{+}=\left[\begin{array}{l} Q_{1}-A_{12}A_{22}^{-1}Q_{2}\\ 0 \end{array}\right]$ matrix was obtained by taking the difference between the original group **Q** and modified group **Q**^** + **^contributions. The effect of this is that genotyped animals do not get a group correction (as in Q*), and non-genotyped get only a group correction as far as their genotypes cannot be predicted from the genotyped animals. The derivation of the **Q-Q**^** + **^correction follows that of the derivation of the **J** factor but applied to genetic groups (see Supplementary Appendix B).

### Alternative models for data analysis

We considered several SSGBLUP models for comparing differences in level-bias, inflation, and stability of genomic predictions. The models differ in whether **J** or **Q** (or the **Q** variants: Q*, **Q**^** + **^and **Q-Q**^**+**^) were fitted or not. A summary of the models is given in Table 2. All the methods fitted the general model:

**Table 2.** Summary of model options (t), numbers (#), acronyms, and descriptions

#	Option(t)^1^	Acronym	Brief description
1	—	SSGBLUP_N	A base model that fitted neither **J** nor **Q.**
2	$J\mu_{g}$	SSGBLUP_J	**J** was fitted as a fixed covariate.
3	u	SSGBLUP_Jr	**J** was fitted as random variable via G modification.
4	Qg	SSGBLUP_Q	**Q** was fitted as fixed covariates.
5	Qg +$\;\;\;J\mu_{g}$	SSGBLUP_QJ	**Q** and **J** fitted in the model as fixedcovariates.
6	Qg	SSGBLUP_QJr	Fixed **Q** was fitted with random **J** covariate.
7	$Q^{*}g^{*}$	SSGBLUP_Q*	The **Q** matrix was modified to Q* using J*=**J-1**.
8	$Q_{01}^{*}g_{01}^{*}$	SSGBLUP_$Q_{01}^{*}$	Q* was obtained using **J** values limited to 0 to 1.
9	$\left(Q-Q^{+}\right)g^{-}$	SSGBLUP_Q-Q^+^	The **Q-Q**^**+**^ fits group effects corrected for the part that can be explained by genotypes.
10	${\left(Q-Q^{+}\right)}_{0}g_{0}^{-}$	SSGBLUP_Q-$Q_{0}^{+}$	Minimum value of $Q-Q^{+}$ is set zero
11	Qg	SSGBLUP_EUPG	**Q** was QP transformed and fitted as random variable following Masuda et al (2021) method.

**u** is a vector of random animal effects, which implicitly account for **J** effects; $\mu_{g}$ is effects of **J** covariate; **g**, $g^{*}$, $g_{01}^{*}$, $g^{-}$, or $g_{0}^{-}$ is a vector of genetic group **Q**, Q*, **Q-Q**^**+**^, ${\left(Q-Q^{+}\right)}_{0}$, or $Q_{01}^{*}$ estimates, respectively.


$$\begin{array}{l} y=Xb+Wh+Zu+Z\left(t\right)+e, \end{array}$$

where **y** is a vector of 1^st^ lactation milk yields; **b** is a vector of fixed effects of year × month of calving, age × lactation number, and days open; **h** is a vector of random herd-year effects; **u** is a vector of random animal effects; **t** denotes model options for fitting **J** or **Q** or variants of **Q** effects, which are described below**; e** is a vector of random residual effects. **X**, **W**, and **Z** are design matrices that relate records to the corresponding effects.

In this study, eleven model options **t** were fitted and evaluated under different scenarios. For the first model, the model option **t** does not exist as this model is a base model for comparison that fitted neither **J** nor **Q** (SSGBLUP_N). This model is not relevant in practice as corrections for genetic groups and base population differences are needed. In SSGBLUP_N, GEBV are the same as the animal solutions. For the second model, $t\;=\;\;\;\;J\mu_{g}$ and **J** was fitted as a fixed covariate with $\mu_{g}$effect, and the model is denoted as SSGBLUP_J. In the SSGBLUP_J model, GEBV are defined as $\hat{u}+J{\hat{\;}\mu \;}_{g}$. For the third model, **t** = **u** and **J** was implicitly fitted as a random variable (SSGBLUP_Jr) following Vitezica et al. (2011)’s method, i.e., the mean difference between the **A**_**22**_ and **G** matrices was added to all elements of the **G** matrix. The first **Zu** term in the above general model is not needed for the SSGBLUP_Jr model. Here, GEBV = $\hat{u}$, which implicitly accounted for the **J** effect. For the fourth model, **t** = **Qg** and **Q** was fitted as fixed covariates (SSGBLUP_Q) with group effect **g**. This model is relevant as it fits genetic groups, but it failed to correct for genetic base differences between **A** and **G**. Here, GEBV = $\hat{u}+Q\hat{g\;}$.

In addition to fitting **J** and **Q** separately, we have fitted **Q** and **J** jointly in the same model in several ways. In the fifth model, $t=Qg+J\mu_{g}$, where **Q** and **J** were fitted simultaneously as fixed covariates (SSGBLUP_QJ). Here, **J** and **Q** were assumed independent and have separate effects. $GEBV=\hat{u}+Q\hat{g}+J{\hat{\;}\mu \;}_{g}.$ For the sixth model, **t** = **Qg**, where **Q** was fitted as fixed covariate together with an implicit random **J** covariate (SSGBLUP_QJr). GEBV = $\hat{u}+Q\hat{g\;}$, and here, the $\hat{u}$implicitly accounted for the **J** effect by modifying the **G** matrix. For the seventh model **t** = Q*g*, where the **Q** matrix was modified to Q* and fitted as fixed covariates (SSGBLUP_Q*) with g* effects. Here, GEBV was defined as $\hat{u}+Q^{*}{\hat{g}}^{*}$. The **J** values are generally between 0 (for non-genotyped animals whose genotyped cannot be predicted) and 1 (for genotyped animals), but we observed values beyond this range (Table 1). So, in the eighth model (SSGBLUP_$Q_{01}^{*}$) where $t=Q_{01}^{*}g_{01}^{*},J$ values were truncated to be between 0 and 1 (negative **J** values were set to 0 while **J** values greater than 1 were set to 1), and then Q* was obtained as in the seventh model but using the truncated **J** values. Here, $GEBV=\hat{u}+Q_{01}^{*}{\hat{g}}_{01}^{*}$, where ${\hat{g}}_{01}^{*}$ are estimated group effects.

The ninth model was to fit $t=\;\left(Q-Q^{+}\right)g^{-}$. The **Q-Q**^** + **^fits group effects corrected for the part that can be explained by the genotype data (SSGBLUP_Q-Q^+^). Here, GEBV= $GEBV=\hat{u}+\left(Q-Q^{+}\right){\hat{g}}^{-}$, where ${\hat{g}}^{-}$ are predicted group effects. Minimum values of **Q-Q**^** + **^are expected to be non-negative but negative values were observed in practice. Hence, effects of imposing restrictions on **Q-Q**^** + **^values to non-negative number (**Q-Q**^** + **^values less than zero were set to 0) were evaluated in the tenth model $\left(SSGBLUP\_Q-Q_{0}^{+}\right)$. For this model, $t={\left(Q-Q^{+}\right)}_{0}g_{0}^{-}$. Here, GEBV was defined as $\hat{u}+{\left(Q-Q^{+}\right)}_{0}{\hat{g}}_{0}^{-}$, where ${\hat{g}}_{0}^{-}$ were predicted group effects. Finally, **Q** was QP transformed and fitted as random variable following Masuda et al. (2021)’s EUPG method (SSGBLUP_EUPG). Here, GEBV = $\hat{u}$, which also include genetic group effects. The EUPG method first includes the group effects into the **A** matrix by the QP transformation resulting in $A_{\Sigma }^{*}$, and next uses the single-step procedure to calculate $H^{*}$ (see below for details), instead of the other way around, i.e., first calculate **H**^**−1**^ and next include group effects [as in Misztal et al. (2013)]. Thereby, EUPG assumes that genomic relationships improve upon pedigree relationships that include group effects, instead of improving upon pedigree relationships before including group effects (Masuda et al., 2021).

All the SSGBLUP models were used as implemented in DMU (Madsen and Jensen, 2013) without using the G-ADJUST option except for SSGBLUP_EUPG model where for computational reasons, MiX99 (Lidauer et al., 2019) was used for prediction. Genomic breeding values were predicted using variance components ($\sigma_{a}^{2}=\;0.250$, additive genetic variance;$\;\;\sigma_{h}^{2}=0.346$, herd-year variance; and $\sigma_{e}^{2}=0.699$, residual variance) and heritability (*h*^2^ = 0.263). This is a single lactation version of the variance components used in GENO’s routine evaluation, where the variance explained by permanent environment variance is transferred to the residual variance.

### Genomic relationships and inverse of unified relationship matrix

The SNP markers were used to construct the **G** matrix as in VanRaden (2008) using the program Gmatrix v2 (Su and Madsen, 2014) i.e., $G=\frac{MM^{\prime }}{\underset{j}{\sum }2p_{j}\left(1-p_{j}\right)},$ where **M** is a matrix of standardized genotypes with elements M_ij_ denoting the number of 1 alleles of animal i at marker j expressed as a deviation from its mean, 2p_j._ The allele frequency p_j_ was calculated based on observed genotypes. To make **G** invertible, a value of 0.01 was added to its diagonal elements. For the SSGBLUP_Jr, SSGBLUP_QJr, and SSGBLUP_EUPG models, the **G** matrix was scaled to correct for genetic base differences between genotyped and non-genotyped individuals as $G^{*}=G+11^{\prime }a$, where **a** is a constant value that was calculated as the mean difference between **A**_**22**_ and **G** (Vitezica et al., 2011). The inverse of the combined relationship matrix was implicitly constructed by DMU (Madsen and Jensen, 2013) as $H^{-1}=\left[\begin{array}{cc} A^{11} & A^{12}\\ A^{21} & A^{22}+GRM^{-1}-A_{22}^{-1} \end{array}\right]$ (Legarra et al., 2009; Aguilar et al., 2010; Christensen and Lund, 2010), where **GRM** is the genomic relationship matrix, which is based on either **G** or G^*^. The inverse of numerator relationship matrix is $A^{-1}=\left[\begin{array}{cc} A^{11} & A^{12}\\ A^{21} & A^{22} \end{array}\right]$. For SSGBLUP_EUPG, we constructed the **H**^**−1**^ following Masuda et al. (2021)’s EUPG method using their Equation (6) as $H^{*}=A_{}^{*}+\left[\begin{array}{ccc} 0 & 0 & 0\\ 0 & {G^{*}}^{-1}-A_{22}^{*} & 0\\ 0 & 0 & 0 \end{array}\right]$. The $A_{\Sigma }^{*}$ is inverse of the numerator relationship matrix including random genetic group effects using the QP transformation (Quaas and Pollak, 1981) and was computed as$A_{\Sigma }^{*}=\left[\begin{array}{cc} A^{-1} & -A^{-1}Q\\ -Q^{\prime }A^{-1} & Q^{\prime }A^{-1}Q+\Sigma^{-1} \end{array}\right],$ where **Ʃ** is the additive relationship among group effects. Following Masuda et al. (2021), we assumed that **Ʃ** is the identity matrix. The ${G^{*}}^{-1}$ is inverse of the scaled **G** matrix ($G^{*}$). The $H^{*}$ matrix and all other matrices required for construction of $H^{*}$were computed in Julia (Bezanson et al., 2017). The $H^{*}$ was provided to MiX99 (Lidauer et al., 2019) for genomic prediction in GBLUP setting, and results from the analyses with $H^{*}$ were compared to those with **H**^**-1**^ where groups were fitted as fixed covariates.

### Evaluation of the models

Inflation, level-bias, and stability of GEBV from the alternative models were estimated using the linear regression (LR) method (Legarra and Reverter, 2018; Macedo et al., 2020a). The LR method estimates these parameters based on two subsets of GEBV that are estimated with “partial” (less information) and “whole” (more information) datasets for the same individuals. This method relies on the assumption that $cov\left({\hat{u}}_{w},{\hat{u}}_{p}\right)=Var\left({\hat{u}}_{p}\right),$ where ${\hat{u}}_{p}$ and ${\hat{u}}_{w}$ denote the GEBV based on the partial and whole data set, respectively. Reverter et al. (1994) showed that this assumption is valid when additional phenotypes become available when moving from the partial to the whole data set. Supplementary Appendix C shows that the ($Cov\left({\hat{u}}_{w},{\hat{u}}_{p}\right)=Var\left({\hat{u}}_{p}\right)$ assumption also holds when extra genotypes or extra pedigree information become available when moving from the partial to the whole data set.

In this study, four partial datasets (scenarios) were obtained by masking phenotypes (GPed-only scenario), or genotypes (PPed-only scenario), or both phenotypes and genotypes (Ped-only scenario), or pedigree info (NoPed scenario) for 674 young validation animals. The whole dataset contains all available genotypes, phenotypes, and pedigree information (GPPed). Information considered or excluded in each scenario is summarized in Table 3. The GPed-only scenario, where only genotype and pedigree information were used, is mimicking the prediction of genotyped bulls and heifers/cows without phenotype. The PPed-only scenario, where only phenotypes and pedigree data were used, is mimicking the prediction of non-genotyped animals with phenotypes. The Ped-only scenario, where only pedigree information was used, is representing animals without any phenotypic and genotypic information, which is the case for many animals in the pedigree. In the NoPed scenario in which parents of the validation individuals were set to missing, **J, Q,** and **Q** variants (Q*, **Q**^** + **^and **Q-Q**^+^) were re-calculated. In this case, analyses were conducted using the genotype, phenotype, and new **J** or **Q** contributions, and the pedigree with missing parents.

**Table 3.** Information included (x) or excluded (−) in the analysis for the 674 animals constituting the validation population in the scenarios considered

Scenario	Phenotypes	Pedigree	Genotypes
GPPed	x	x	x
GPed-only	−	x	x
PPed-only	x	x	−
Ped-only	−	x	−
NoPed	x	−	x

Using the LR method, we calculated an estimator for inflation (${\hat{b}}_{p}$), for level-bias $\left({\hat{\Delta }}_{p}\right)$ and stability of GEBV $\left({\hat{\;}\rho \;}_{w,p}\right)$. A summary of the estimators is given below (see Legarra and Reverter, 2018; Macedo et al., 2020a, b for details).

#### Inflation (${\hat{b}}_{p}$)

The estimator of inflation of GEBV was measured as the regression of ${\hat{u}}_{w}$ on ${\hat{u}}_{p}$, i.e.,${\hat{b}}_{p}=\frac{cov\left({\hat{u}}_{p},\;\;\;{\hat{u}}_{w}\right)}{var\left({\hat{u}}_{p}\right)}$. The expected value of ${\hat{b}}_{p}$ is 1. Values of ${\hat{b}}_{p}<1$ indicate over-dispersion and ${\hat{b}}_{p}>1$ indicate under-dispersion of GEBV.

#### Level-bias (${\hat{\Delta }}_{p}$)

The estimator of level-bias was measured as the difference in means between the ${\hat{u}}_{p}$ and ${\hat{u}}_{w}$ and was scaled by the genetic standard deviation of milk yield, $\sigma_{a}$=0.5, which is common for all models as ${\hat{\Delta }}_{p}=\frac{mean\left({\hat{u}}_{p}-{\hat{u}}_{w}\right)}{\sigma_{a}}$. In the absence of bias, the expected value of ${\hat{\Delta }}_{p}$ is zero.

#### Ratio of accuracies or stability (${\hat{\rho }}_{w,p}$)

Ratio of accuracies (Legarra and Reverter, 2018) or stability of GEBV (Kluska et al., 2021) was measured as the correlation between ${\hat{u}}_{p}$ and ${\hat{u}}_{w}$. The stability of GEBV measures consistency between GEBVs from two subsequent evaluations (Kluska et al., 2021). This estimator estimates the inverse of relative gain in accuracy due to addition of information to partial datasets, i.e., relative increase in accuracy from ${\hat{u}}_{p}$ to ${\hat{u}}_{w}$ or in stability of the GEBV when moving from partial to whole data sets.

## Results

### Descriptions and effects of the J covariate

Table 1 shows descriptive statistics of **J** covariate for all animals in pedigree. There were marginal differences between the two sets of **J** values computed with and without setting parents of validation animals to missing as far as the values for all animals in pedigree were concerned, but there were some differences between the two sets for the validation animals. Although genotyped animals have a **J** value of 1 and non-genotyped animals unrelated to the genotyped animals have a **J** value of 0, not all **J**-values were within the range between 0 and 1. In our data, 444,017 animals have J > 1 while 18,750 animals have J > 1.1. Looking at the most extreme values, 81 animals have J > 1.3 and the largest J was 1.676. Most of these 81 animals are old bulls, born before 1978 except three of them that were born in 2013 and 2014 with 5 to 8 offspring. These old bulls may have many genotyped offspring or have many genotyped descendants. Few animals had negative **J** values: 88 animals have J < −0.0001 and the smallest J is −0.014. These 88 animals are all old cows, born mostly before 1974 (the youngest born in 1989). These cows might have been mated to those old bulls and are dams (granddams) of some of the oldest genotyped bulls.

Estimates of the **J** covariate effects $\left(\mu_{g}\right)$ from the different genomic prediction models (Table 2) under the various scenarios (Table 3) are given in Table 4. The effects of the **J** covariate differed between the models but were similar across scenarios within a model. Compared to the estimates in the SSGBLUP_J model, higher estimates of **J** effects were found when **Q** was fitted together with the **J** factor. This indicates that fitting **J** together with genetic groups would have larger impact on the GEBV than fitting the **J** covariate alone.

**Table 4.** Regression coefficient estimates of **J** covariate effects from the different genomic prediction models under various scenarios

Model	Scenario^1^				
	GPPed	GPed-only	PPed-only	Ped-only	NoPed
SSGBLUP_J	0.834	0.833	0.830	0.829	0.833
SSGBLUP_QJ	2.619	2.620	2.614	2.614	2.543

Scenarios are as described in Table 3.

### Trends for genetic group effects

**Figure 2.**
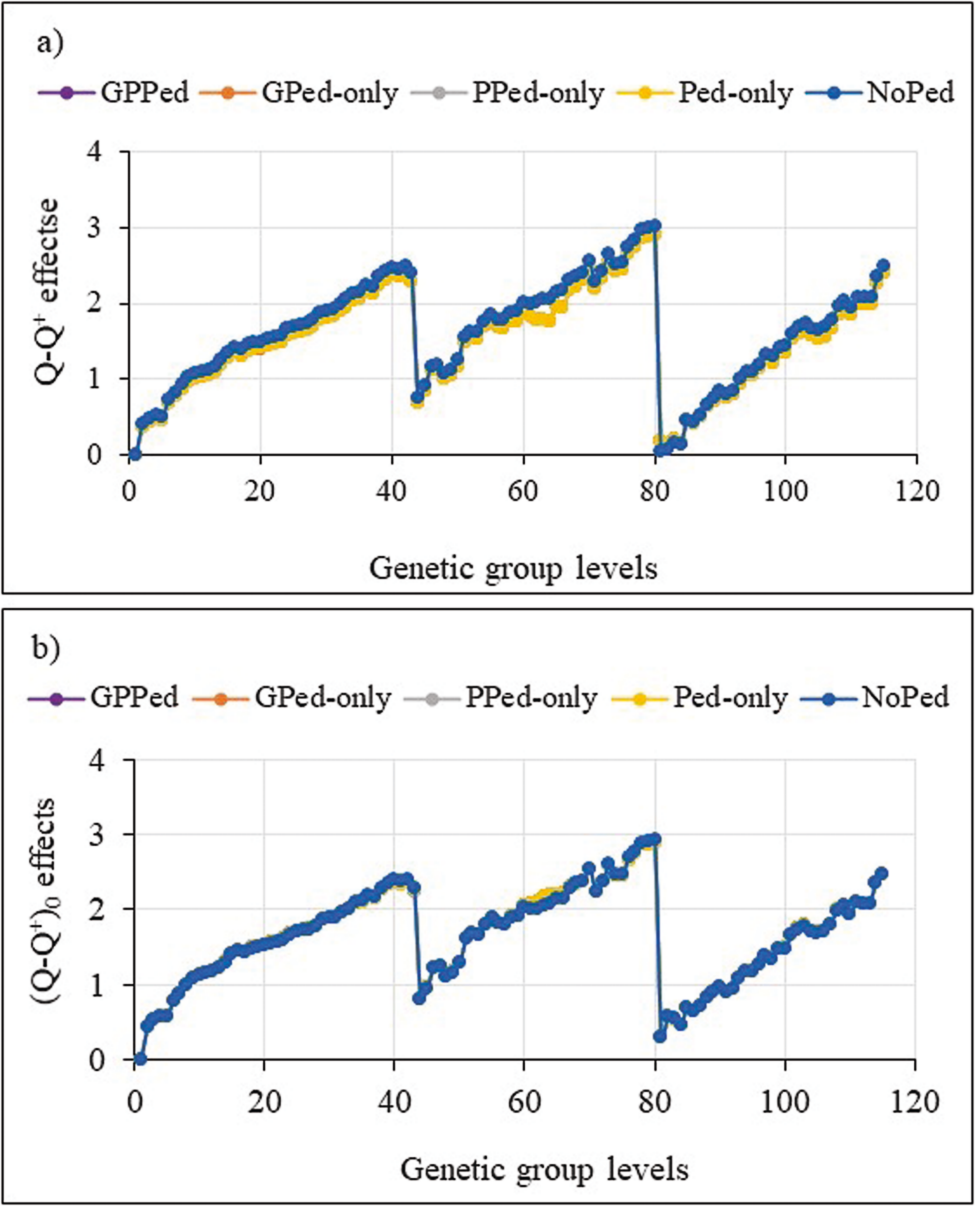
Trends for the original Q-Q^+^ (SSGBLUP_Q-Q^+^) (a) and restricted Q-Q^+^ (where minimum value of the Q-Q^+^ set to zero: $SSGBLUP\_Q-Q_{0}^{+}$) (b) effects estimated using the partial datasets (where phenotypes (GPed-only), or genotypes (PPed-only), or both phenotypes and genotypes (Ped-only), or pedigree information (NoPed) of the 674 cows masked) and whole dataset (GPPed).

**Figure 3.**
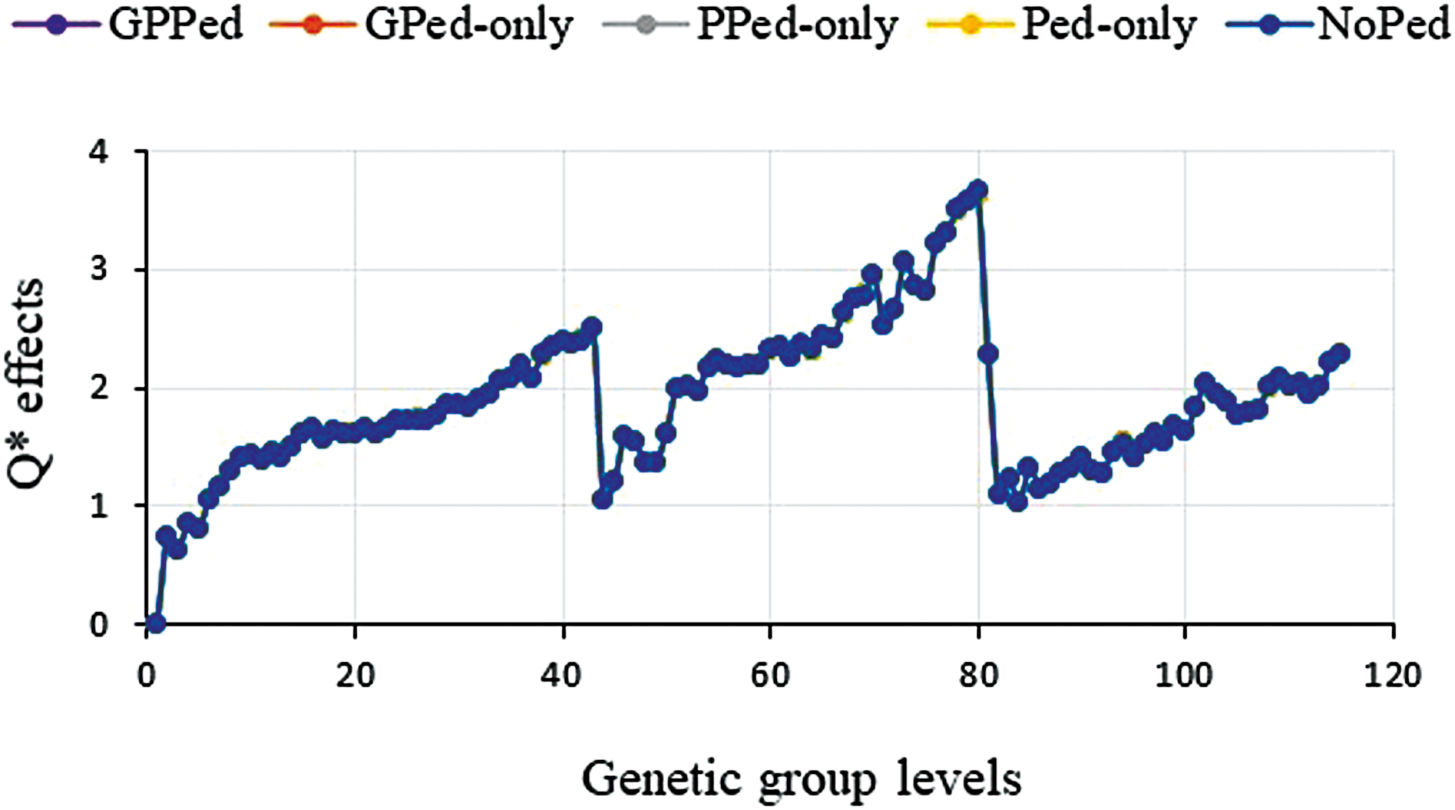
Trends for Q* (SSGBLUP_Q*) effects estimated using partial datasets (where phenotypes (GPed-only), or genotypes (PPed-only), or both phenotypes and genotypes (Ped-only), or pedigree information (NoPed) of the 674 cows masked) and whole dataset (GPPedd).

**Figure 4.**
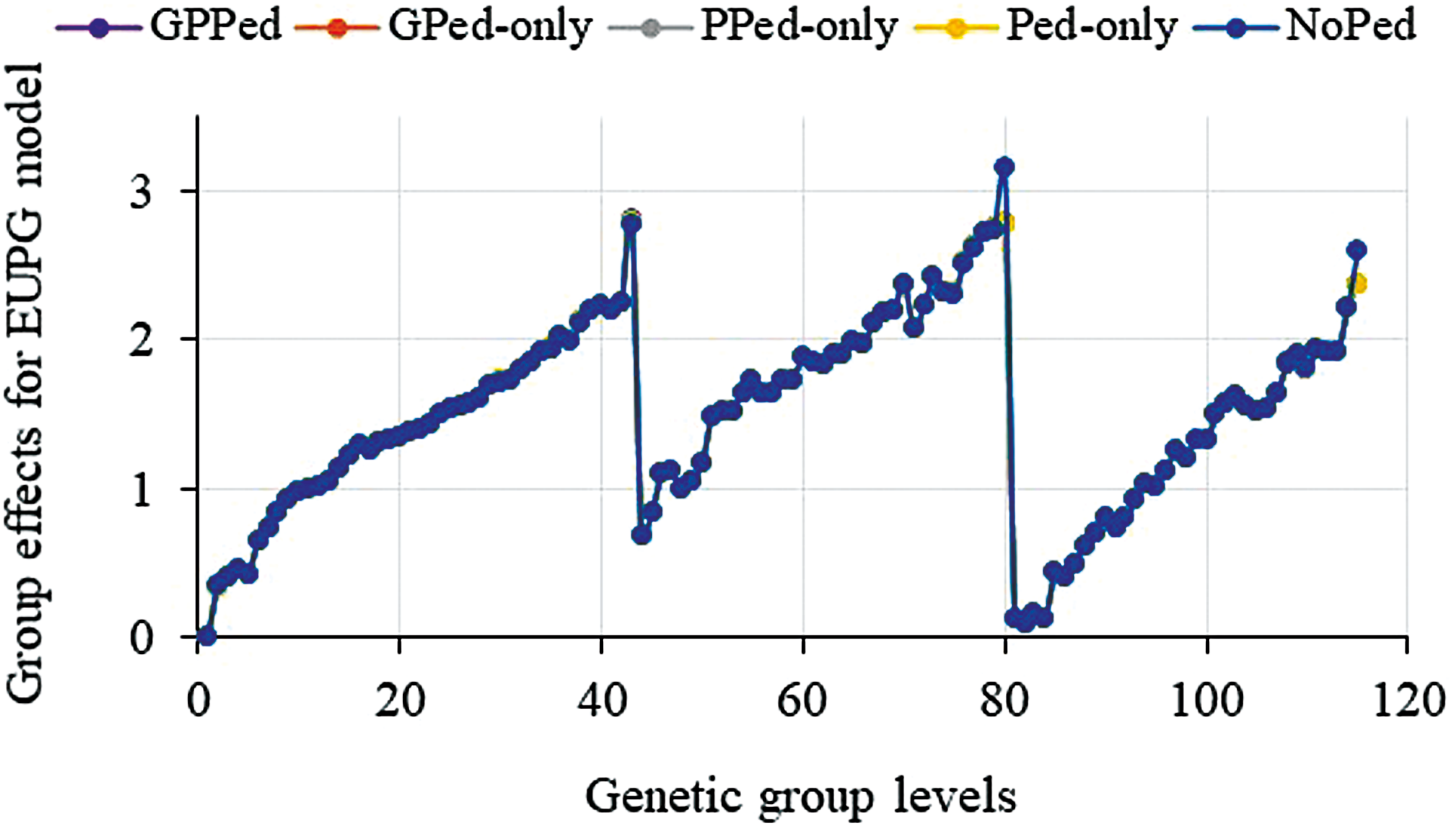
Trends for the QP transformed genetic groups (SSGBLUP_EUPG) effects estimated using partial datasets (where phenotypes (GPed-only), or genotypes (PPed-only), or both phenotypes and genotypes (Ped-only), or pedigree information (NoPed) of the 674 cows masked) and whole dataset (GPPedd). Note: Trends for genetic group effects shown in Figures 2–4 were adjusted by the first group prediction in each model and prediction scenario.

Trends for genetic group effects are shown in Figures 2–4, which were adjusted by the first group prediction in each model and prediction scenario. We show only results for the most promising models and omitted some models due to similarities of the results. In Figures 2–4, the jumps in trends for genetic group effects around levels 43 and 80 are due to sorting of the groups by year within each category of missing parents, which is a missing on-farm bull or a missing AI sire or a missing dam. Models with the **Q-Q**^** + **^contributions overestimated trends for group effects in the scenario with missing pedigree (NoPed in Figure 2a). In the other scenarios, genetic group effects were marginally overestimated. By setting the minimum values of the **Q-Q**^** + **^contributions to zero, the differences in genetic group predictions that were observed in Figure 2a for the scenario with missing pedigree were greatly reduced and the model achieved similar trends in all the scenarios (Figure 2b). Like other models, the model with Q* gave increasing trends for group effects but had group estimates above one for all group levels in the last two categories of missing parents (Figure 3). The model with truncated Q* values (i.e., setting **J** values between 0 and 1, then using them to compute Q*) achieved very similar results as the model with Q* (results not shown). The model with QP transformed **Q** that fitted random group effects gave similar genetic group predictions (Figure 4). Genetic groups predictions were nearly unbiased for all models across scenarios, in the sense that the missing data hardly affected the trend estimates. The exception was in the missing pedigree scenario where trends for group effects were slightly overestimated (plots for NoPed are on top of the other scenarios for Figures 2–4). The models achieved similar trends for genetic group effects in all the scenarios.

### Inflation of GEBV

Estimates of inflation (${\hat{b}}_{p}$) in GEBV for the alternative prediction models under the different scenarios are presented in Table 5. For a given scenario, differences between the models were small, and the models achieved similar inflation. In the scenarios with genomic information, fitting **J** either as fixed or random together with or without **Q** resulted in GEBV with similar inflation. However, in the scenarios with missing genomic information, GEBV were relatively more inflated when **J** was fitted as random together with or without **Q** compared to fitting it as fixed variable. Unlike other models where **J** and **Q** were fitted in alternative forms, models with Q* or **Q-Q**^** + **^achieved the lowest inflation in the scenario with missing pedigree (Table 5). The model with QP transformed **Q** (SSGBLUP_EUPG) performed similarly to models with Q* or **Q-Q**^**+**^. Imposing restrictions on $J\left(SSGBLUP\_Q_{01}^{*}\right)$ or $Q-Q^{+}\left(SSGBLUP\_Q-Q_{0}^{+}\right)$ values had marginal effects on inflation except for the **Q-Q**^** + **^model where inflation was reduced to 1.001 in the NoPed scenario (Table 5).

**Table 5.** Regression coefficients (as measure of inflation) of GEBV from the whole dataset on GEBV from the partial datasets (scenarios) for validation animals and their standard errors (in parenthesis) using the alternative models

Model	Scenario^1^			
	GPed-only	PPed-only	Ped-only	NoPed
SSGBLUP_N	0.997(0.008)	0.921(0.028)	0.932(0.037)	1.003(0.003)
SSGBLUP_J	1.007(0.008)	0.946(0.025)	0.973(0.034)	1.005(0.003)
SSGBLUP_Jr	1.007(0.008)	0.942(0.026)	0.960(0.034)	1.005(0.003)
SSGBLUP_Q	1.009(0.008)	0.928(0.025)	0.946(0.034)	0.991(0.004)
SSGBLUP_QJ	1.001(0.007)	0.970(0.025)	0.990(0.033)	1.022(0.009)
SSGBLUP_QJr	1.001(0.007)	0.955(0.028)	0.966(0.037)	1.022(0.009)
SSGBLUP_Q*	1.007(0.008)	0.947(0.025)	0.971(0.034)	1.004(0.003)
$SSGBLUP\_Q_{01}^{*}$	1.008(0.008)	0.945(0.025)	0.970(0.034)	1.004(0.003)
SSGBLUP_Q-Q^+^	1.006(0.008)	0.947(0.026)	0.968(0.034)	1.003(0.003)
$SSGBLUP\;\_\;Q\text{-}Q_{0}^{+}$	1.008(0.008)	0.943(0.025)	0.965(0.034)	1.001(0.029)
SSGBLUP_EUPG	1.006(0.008)	0.947(0.026)	0.962(0.035)	1.005(0.003)
Average	1.005(0.008)	0.945(0.026)	0.964(0.034)	1.006(0.006)

Scenarios are as described in Table 3.

When comparing scenarios across the models, inflation was virtually absent for genotyped young animals (on average, ${\hat{b}}_{p}=1.006$ for the GPed-only and NoPed scenarios) compared with estimates for non-genotyped young individuals (0.957 for the PPed-only and Ped-only scenarios). Hence, inflation of GEBV is reduced by genotyping individuals. For example, inflation was reduced by 0.04 points on average due to genotyping animals with only pedigree information (GPed-only vs. Ped-only) while phenotyping them increased it by −0.02 points (PPed-only vs. Ped-only). Therefore, inflation was reduced by 0.06 points on average due to genotyping relative to phenotyping animals that only had pedigree information (GPed-only vs. PPed-only). The most inflated GEBV were observed in the situation where both phenotypes and pedigree information (PPed-only) of the young animals was used for prediction. Also, in the scenarios with missing genotypic information, GEBV were inflated since ${\hat{b}}_{p}$ was less than one.

### Level-biases of GEBV

Table 6 shows scaled mean differences between breeding values (level biases) from whole and partial datasets analyses for the different models. In the scenarios with genomic information (GPed-only and NoPed), effects of fitting the **J** factor as random together with or without **Q** on level-biases were similarly to fitting it as a fixed covariate. However, in the scenarios with missing genotypic information (PPed-only and Ped-only), fitting the **J** factor as a fixed covariate together with or without **Q** resulted in less biased GEBV compared to treating it as a random variable (Table 6). Therefore, treating the **J** factor as a fixed covariate is recommended to reduce level-bias.

**Table 6.** Level-biases and standard errors (in parenthesis) of GEBV estimated as mean differences (in genetic standard deviations) between GEBV from whole and partial (scenarios) datasets using the alternative models

Model	Scenario^1^			
	GPed-only	PPed-only	Ped-only	NoPed
SSGBLUP_N	−0.041(0.008)	0.197(0.026)	0.139(0.029)	−0.021(0.003)
SSGBLUP_J	−0.024(0.008)	−0.125(0.024)	−0.178(0.028)	−0.016(0.003)
SSGBLUP_Jr	−0.024(0.008)	−0.145(0.024)	−0.201(0.028)	−0.017(0.003)
SSGBLUP_Q	0.031(0.008)	0.033(0.023)	−0.022(0.027)	0.181(0.004)
SSGBLUP_QJ	0.040(0.008)	−0.081(0.024)	−0.082(0.028)	1.119(0.010)
SSGBLUP_QJr	0.039(0.008)	−0.137(0.026)	−0.152(0.030)	1.100(0.010)
SSGBLUP_Q*	−0.022(0.008)	−0.099(0.023)	−0.149(0.027)	−0.014(0.009)
$SSGBLUP\_Q_{01}^{*}$	−0.023(0.008)	−0.086(0.023)	−0.137(0.027)	−0.014(0.009)
SSGBLUP_Q-Q^+^	−0.023(0.008)	−0.089(0.024)	−0.141(0.028)	−0.012(0.003)
$SSGBLUP\;\_\;Q-Q_{0}^{+}$	−0.023(0.008)	−0.070(0.023)	−0.121(0.028)	−0.009(0.003)
SSGBLUP_EUPG	−0.033(0.008)	−0.131(0.023)	−0.187(0.028)	−0.082(0.003)
Average	−0.009 (0.008)	−0.067(0.024)	−0.112(0.028)	0.201(0.005)

Scenarios are as described in Table 3.

In the scenario with missing phenotypes (GPed-only), the differences in level bias between the models were marginal and the models achieved similar level biases, which were not significantly (*P* > 0.05) different from zero. However, in the remaining scenarios, the differences in level bias between most models were considerable and the level biases were significantly (*P* < 0.05) different from zero. Some models such as SSGBLUP_Q and SSGBLUP_QJ performed better in the first three scenarios in which the level biases were both not significantly different from zero and between the scenarios for a given model. In the fourth scenario (NoPed), however, level-bias was higher for those and another model (e.g., SSGBLUP_QJr) where **Q** was fitted. Such higher level-biases were greatly reduced when there was a correction for the part of the genetic group that could be explained from the genotypes in the form of Q* or **Q-Q**^**+**^ in the scenario with missing pedigree. Fitting QP transformed **Q** as random resulted in higher estimates of level-bias than in the models with Q* or **Q-Q**^**+**^. Imposing restrictions on **J** or **Q-Q**^**+**^ values further reduced level-biases for models with Q* or **Q-Q**^** +**^ (Table 6). Among these competent models, the models with **Q-Q**^**+**^ (either with original or truncated values) were performing slightly better than corresponding models with Q*. Moreover, **Q-Q**^**+**^ has theoretical justification (see Supplementary Appendix B).

For most models, GEBVs were more biased in the Ped-only than in the NoPed scenario (Table 6). However, this is not true when average biases across models were calculated due to the extremely high bias estimates for two models (SSGBLUP_QJ and SSGBLUP_QJr), which is reflected in these averages. Hence, GEBVs were mostly biased in the scenario with missing pedigree (${\hat{\Delta }}_{p}=0.201$ on average) and followed by the scenario where only pedigree information was used for prediction (${\hat{\Delta }}_{p}=-0.112$). The GEBVs were least biased in the scenario where only relationship matrixes (both **A** and **G**, i.e., GPed-only) were used for prediction (${\hat{\Delta }}_{p}=0.009$).

It may be interesting to consider animals without phenotypes nor genotypes (Ped-only) and to study the effect on level-biases when either phenotyping or genotyping these animals. This is revealed by comparing the GPed-only with Ped-only scenarios, versus the PPed-only with Ped-only scenario. Reduction in level-biases due to genotyping of animals with only pedigree information ranged from 0.191 to -0.180, with an average of 0.102 points (GPed-only vs. Ped-only). The corresponding values for phenotyping of such individuals ranged from -0.001 to 0.058, with an average of 0.045 points (PPed-only vs. Ped-only). Therefore, level-biases are more reduced by genotyping than by phenotyping the animals (by 0.057 points on average).

In most models, the sign of the level-biases was negative indicating that GEBV means for validation animals in partial datasets were lower than those in whole datasets. Hence, GEBVs generally moved upwards when more data become available.

### Stability of GEBV

The stability of GEBV that measured as the correlation between GEBV from whole and partial datasets is presented in Table 7. Generally, differences in stability between the models were small (Table 7), and the models achieved similar stability of GEBV in each of the scenarios. In the scenarios with genomic information, there was no difference in stability of GEBV whether fitting **J** as fixed or random. In the scenarios with missing genomic information, however, fitting **J** as a fixed covariate particularly together with **Q** (SSGBLUP_QJ) improved the stability significantly (*P* < 0.05, based on Fisher’s *z*-transformation of correlation coefficients) compared to fitting it as a random in the corresponding model, SSGBLUP_QJr. The models with Q* or **Q-Q**^**+**^ achieved relatively a higher stability of GEBV. Fitting QP transformed **Q** as random achieved similar stability as in the models with Q* or **Q-Q**^**+**^. Imposing restrictions on **J** or **Q-Q**^**+**^ values had marginal effects on stability of GEBV except for the **Q-Q**^**+**^ model where the stability was improved in the Ped-only scenario.

**Table 7.** Stabilities or correlations between GEBV from whole and partial datasets (scenarios) for validation animals using the alternative models

Model	Scenario^1^			
	GPed-only	PPed-only	Ped-only	NoPed
SSGBLUP_N	0.979	0.784	0.701	0.997
SSGBLUP_J	0.980	0.821	0.741	0.997
SSGBLUP_Jr	0.980	0.817	0.733	0.997
SSGBLUP_Q	0.979	0.815	0.728	0.995
SSGBLUP_QJ	0.982	0.829	0.757	0.974
SSGBLUP_QJr	0.982	0.798	0.713	0.975
SSGBLUP_Q*	0.981	0.821	0.740	0.997
$SSGBLUP\_Q_{01}^{*}$	0.980	0.822	0.740	0.997
SSGBLUP_Q-Q^+^	0.981	0.818	0.736	0.997
$SSGBLUP\;\_\;Q-Q_{0}^{+}$	0.981	0.819	0.756	0.997
SSGBLUP_EUPG	0.981	0.816	0.731	0.997
Average	0.980	0.815	0.734	0.993

Scenarios are as described in Table 3.

There were significant (*P* < 0.001) differences in stability of GEBV between the scenarios within a model except between GPed-only and NoPed scenarios. On average, the estimates were highest in the scenario with missing pedigree information (${\hat{\;}\rho \;}_{w,p}=0.99$), and lowest for the scenario where only pedigree information (${\hat{\;}\rho \;}_{w,p}=0.73$) was used. Stability of GEBV was improved on average by 0.246 points (33%) due to genotyping of animals with only pedigree information (GPed-only vs. Ped-only). However, it was improved only by 0.08 points (11%) due to phenotyping of these individuals (PPed-only vs. Ped-only). Hence, stability of GEBV was improved on average by 0.163 points (20%) due to genotyping over phenotyping of animals with only pedigree information (GPed-only vs. PPed-only).

## Discussion

In this study, the **J** factor and **Q** contributions were derived and fitted as a fixed or random variable to evaluate their effects on inflation, level-bias, and stability of GEBV in the SSGBLUP model using milk production data from Norwegian Red cattle. Estimates for level-bias, inflation, and stability of GEBV were obtained using the LR method (Legarra and Reverter, 2018) and with evaluation models that fitted different sources of information (scenarios) with different strategies to model genetic groups and to overcome base-population differences between **A** and **G**.

### Effects of J covariate on genomic predictions

The effect of the **J** covariate can be explained from different perspectives. If all animals are genotyped, fitting $J\mu_{g}$ is like fitting an overall mean, and thus, $\mu_{g}$ is confounded with the overall mean and is redundant. Hence, in GBLUP models, where all animals are genotyped, we do not need to fit a **J** covariate as long as we fit an overall mean. If the genotyped animals are unrelated to the non-genotyped animals, $A_{12}=0$, imputation accuracy is 0 and $J={\left[\begin{array}{ll} 0^{\prime } & 1^{\prime } \end{array}\right]}^{\prime }$. Here, the imputation residual ϵ models the full genetic value of the non-genotyped animals using pedigree relationships in SSGBLUP and accounts for a possible genetic difference between the non-genotyped and genotyped animals, which may be due to differences between the founder populations of the **A** and **G** matrices. Thus, $J\mu_{g}$ can account for a difference in genetic base between **A** and **G**, and as such is also relevant for SSGBLUP models. This could be important in selected populations as differences in means between base populations differing in time may be large in selected populations. In the more common situation where genotyped and non-genotyped animals are related, the non-genotyped animals are modeled by a combination of marker effects (the part that can be predicted from the marker genotypes), and a pedigree-based animal effect, ϵ. The **J** covariate here accounts for the fraction that can be explained by the markers using **A**, which is $A_{12}A_{22}^{-1}1$. The above arguments are also relevant for SSGBLUP models, i.e., the SSGBLUP model should also correct for differences in genetic level that may arise due to base population differences between the **G** and **A** matrices.

Fitting **J** either as fixed or random in the SSGBLUP model generally reduced level biases and inflation and increased stability of GEBV compared to the basic model where neither **J** nor **Q** was fitted (SSGBLUP_N). This agrees with reports from simulation studies that fitted equivalent models in SSGBLUP (Vitezica et al., 2011; Bermann et al., 2021) and ssSNPBLUP (Hsu et al., 2017) methods. However, effects of the **J** factor on inflation of GEBVs were very marginal in the study by Hsu et al. (2017).Vitezica et al. (2011) indicated that implicitly fitting **J** as a random variable is equivalent to explicitly fitting it as a regression coefficient whose covariate is **J**. However, results from the current study indicate that fitting the **J** factor as random variable was less effective than fitting it as a fixed covariate especially in the situation where validation animals have no genotypes (PPed-only) or both missing genotypes and phenotypes (Ped-only). In contrast, Bermann et al. (2021) reported that fitting **J** as fixed performed worse than fitting it as random. These authors however indicated that the bias is likely to be lower in real livestock data as the selection pressure and accuracy of prediction are lower in livestock than in simulated data. Moreover, the discrepancy is partly attributed to differences in data structure (type and size), as well as to how **J** was fitted as fixed (QP transformed or not). Fitting **J** as random together with **Q** also resulted in more biased and lower stability of GEBVs than fitting it as fixed together with **Q**. In addition to improving genomic prediction, fitting the **J** factor as fixed effect is flexible and hence it can be combined with other effects such as the **Q** matrix in different alternative forms as was explored in this study.

The reason(s) behind the slightly better predictions observed in models with fixed **J** than in models with random **J** may relate to indirect dependency of the scaling parameter for **G** (alpha in Vitezica et al., 2011) on base population allele frequency that may not be estimated accurately (Misztal et al., 2020), whereas the fixed version of Vitezica et al. (2011)’s method showed not to depend on base allele frequency (Bermann et al., 2021). The impact of the scaling parameter should be small in genomic prediction for genotyped animals when more genotypes are available. However, the scaling parameter may be more severe for non-genotyped animals because the scaled **G** changes the relationships between genotyped and non-genotyped animals in **H** matrix (Legarra et al., 2009). This is evident in this study where **J** was fitted as random especially in the scenarios with missing genotype information i.e., in the models with scaled **G**, the non-genotyped animals (those in PPed-only and Ped-only scenarios) had poor predictions compared to corresponding values when their genotypic information was considered in the analyses (GPed-only and NoPed). Moreover, it may be complicated to obtain good scaling parameter alpha, which involve **A**_**22**_, in populations with incomplete pedigree that may trace back to several base populations and genetic structure (Bermann et al., 2021). Thus, it appears that the absorption of alpha into SSGBLUP in the presence of genetic groups may be more difficult than for populations with complete pedigrees.

### Effects of combining J and genetic groups on genomic predictions

In the alternative ways of combining the **J** factor with **Q, J** was either fitted simultaneously with **Q** assuming independent and separate effects or combined with group contributions. Fitting **J** simultaneously with **Q** (SSGBLUP_QJ) increased the level-bias compared to the model with **Q** alone (SSGBLUP_Q) especially in the last three scenarios of Table 6. This indicates that effects of the **J** and **Q** corrections do not complement each other as there might be confounding issues when they are fitted simultaneously in the same model assuming separate effects. Moreover, in the scenario with missing pedigree, genomic predictions were more biased and less stable for the models where **Q** was fitted alone or simultaneously with **J** than predictions from models without **Q** fitted. Such biased predictions in the scenario with missing pedigree can be related to the large number of genetic groups used in this study, which might lead to inaccurate estimates of group effects. With large number of groups, the number of animals and phenotypic information for each group can be insufficient to accurately estimate group effects, and missing pedigree may amplify bias in group effects (Tsuruta et al., 2014). These authors showed that combining groups with small amount of information helped to reduce GEBV biases in the Holstein population. In setting parents of the validation animals to missing, the relationship between genotyped and non-genotyped animals might become zero (i.e., **A**_**12**_ = 0) and in such situation, **H**^**−1**^ will not contribute to the estimation of group effects (Tsuruta et al., 2019). Truncation of the data set, e.g., only considering data after the year 2000, may also reduce the number of genetic groups, and has been found to reduce prediction biases (Cesarani et al., 2021; Hidalgo et al., 2021; Hollifield et al., 2021; Macedo et al., 2022).

Biased predictions in scenarios with missing pedigree may also be due to fitting genetic group effects in the presence of genomic information, resulting in double counting effects (Masuda et al., 2021). That means the genetic group levels are explained twice in the form of group effects and in the form of genotypic information. Hence, when **J** was used to modify **Q** contributions that resulted in Q*, or **Q-Q**^**+**^, genomic predictions were generally improved compared with predictions from models where **J** and **Q** were fitted simultaneously or separately. Among the models with **Q** variants, the models with **Q-Q**^**+**^ (either with original or truncated values) were performing slightly better across all scenarios at least as far as level-bias is concerned.

Supplementary Appendix D shows the QP transformations of the **Q-Q**^** + **^and Q* models. It may be noticed that the QP transformation of **Q-Q**^**+**^ is the same as the “Altered QP” method (Masuda et al., 2021) where groups are included into $A^{-1}$ and $A_{22}^{-1}$ but not into $G^{-1}$. The only difference is whether group effects were fitted explicitly as regression coefficient (**Q-Q**^**+**^) or included into $A^{-1}$ and $A_{22}^{-1}$ via the QP transformation (Altered QP). Tsuruta et al. (2019) applied the SSGBLUP models with the Altered QP and full QP (when group effects included into $G^{-1}$ and $A_{22}^{-1}$ in addition to $A^{-1}$) to real and simulated data. These authors obtained reduced inflation and improved accuracy in the model with Altered QP compared to full QP in simulated data and found similar results when real data was used. In addition, they observed less bias in genetic trends for both data types. In agreement with our results, Masuda et al. (2019) also observed a reduction in inflation of GEBV with the Altered QP compared with the full QP for type and production traits in US Holstein. Cesarani et al. (2021) showed that the model with Altered QP yielded in more accurate and unbiased evaluations than a model where groups were included only into $A^{-1}$.

Recently, using simulated data, Masuda et al. (2021) evaluated different strategies to model genetic groups in the SSGBLUP method including the EUPG model that we also included in this study using real data. In this study, when phenotype was missing, models with corrected group covariates (e.g., with **Q-Q**^**+**^) performed similarly to the EUPG model with random QP transformed group effects. This agrees with the results of Masuda et al. (2021) who found similar predictive ability (correlation between true breeding values and GEBV) and inflation values in models with Altered QP and EUPG. However, the EUPG model gave a more biased GEBV than the models with Q* and **Q-Q**^**+**^, especially when genomic information was masked in the analyses. On other hand, when the **G** matrix for the EUPG model was not scaled, such biases were basically removed and become like the ones in the models with Q* and **Q-Q**^**+**^ (results not shown in tables). In this study, it seems that scaling the **G** matrix in EUPG model introduced bias which might be related to inaccurate computation of the scaling parameter in population with missing pedigree, as discussed above. The Altered QP method (**Q-Q**^**+**^ in this study) underestimated group predictions in comparison to the EUPG model (Masuda et al., 2021), but such underestimation was not observed in this study. The model with truncated **Q-Q**^**+**^ values performed slightly better than other models across scenarios particularly with regard to level-biases. Masuda et al. (2021) also reported that the EUPG method performed essentially the same as the metafounder approach (Legarra et al., 2015) which has been recommended as the best method for modeling missing parents in the SSGBLUP models as it provided accurate and unbiased predictions compared with other methods for modeling group effects (Bradford et al., 2019; Kudinov et al., 2020; Macedo et al., 2020b).

Fitting the **J** covariate and genetic groups seems a relatively simple way of correcting for differences in genetic means of base populations, which may differ per genetic group. However, this only corrects for the differences in genetic means, and not for other effects that may arise due to differences in base populations. For example, some base populations may be more inbred than others, resulting in a higher level of genetic relationships within this group and a smaller variance of relationships (since relationships are closer to their maximum). Also, some base populations of genetic groups may be more related to each other than others, resulting in increased relationships between all their descendants. The concept of metafounders (Legarra et al., 2015) corrects for all these effects, but is more complicated to implement and requires the estimation of a relationship matrix among the metafounders/genetic groups.

## Conclusions

The effects of the **J** covariate differed between the models but were similar across scenarios within a given model. Trends for genetic group effects were similar for all models, and genetic group predictions were nearly unbiased in all models across scenarios except for NoPed scenario. Fitting **J** as a fixed covariate together with or without **Q** improved genomic predictions when genotypes were missing but performed similarly to fitting it as a random covariate together with or without **Q** when genomic data was included. Level-bias and inflation were reduced, and stability of GEBV were improved for models which fitted Q* or **Q-Q**^**+**^. Imposing restrictions on Q* and **Q-Q**^**+**^ further reduced level-biases but had marginal effects on inflation and stability of GEBV. These models yielded in less level-biases than the model with random QP transformed group effects (EUPG). Any of the models with Q*, **Q-Q**^**+**^ (with or without restricting their values to the 0-1 range) may yield generally unbiased breeding values and genetic group trends. However, models with **Q-Q**^**+**^ were recommended because they showed least bias and highest stability of GEBV across the scenarios [particularly when the minimum (**Q-Q**^**+**^) value was set to 0].

## Supplementary Material

skac227_suppl_Supplementary_Appendix

## Abbreviations

EUPG: encapsulated unknown parent groups
GBLUP: genomic best linear unbiased prediction
GEBV: genomic estimated breeding values
GPed-only: only genotypes and pedigree information were used
GPPed: genotypes, phenotypes, and pedigree information were used
NoPed: no pedigree information (missing pedigree) was used
Ped-only: only pedigree information was used
PPed-only: only phenotypes and pedigree information were used
SNP: single nucleotide polymorphisms
SSGBLUP: single-step genomic best linear unbiased prediction
ssSNPBLUP: single-step SNP best linear unbiased prediction
