# Supplementary material for: Correcting for base-population differences and unknown parent groups in single-step genomic predictions of Norwegian Red cattle
Source: J Anim Sci. 2022 Jun 25;100(9):skac227. doi: 10.1093/jas/skac227 (PMC9467032; doi:10.1093/jas/skac227)
Supplement: skac227_suppl_Supplementary_Appendix [file skac227_suppl_supplementary_appendix.docx]

**APPENDIX A**

***Illustration of the genetic group (Q) variants using an example pedigree***

The **J** factor and **Q** variants were illustrated using a simple pedigree containing 3 animals: $\begin{matrix} 1 & P1 & P2 \\ 2 & 1 & P3 \\ 3 & 1 & 2 \end{matrix}$

where P1, P2 and P3 are missing parents that are grouped into two genetic groups (g_1_ and g_2_) based on sex. The inverse of the numerator relationships matrix among the animals become $\mathbf{A}^{\mathbf{-1}}=\begin{matrix} 1.833 & -0.167 & -1.0 \\ -0.167 & 1.833 & -1.0 \\ -1.0 & -1.0 & 2.0 \end{matrix}$.

Animal number 3 is assumed genotyped. Using this $\mathbf{A}^{\mathbf{-1}}$, one can obtain J =0.6 for the first two non-genotyped animals (Appendix A Table 1), and J=1 is assigned for the genotyped animal. The corresponding values for **J***=**1-J** that used for **Q*** computations are 0.4 and 0 for the non-genotyped and genotyped animals, respectively (results not shown, but it can easily be computed from **J** values presented in the Appendix A Table 1). In this simple pedigree, **J** values are between 0 and 1 unlike the one in real and complex pedigree that used in this study (Table 1).

Additive genetic relationship between the animals (rows) and the missing parents (columns) become **a**=$\left[ \begin{matrix} 0.5 & \begin{matrix} 0.5 & 0.0 \end{matrix} \\ \begin{matrix} 0.25 \\ 0.375 \end{matrix} & \begin{matrix} \begin{matrix} 0.25 \\ 0.375 \end{matrix} & \begin{matrix} 0.5 \\ 0.25 \end{matrix} \end{matrix} \end{matrix} \right]$ and the incidence matrix of missing parents (rows) and the genetic groups (columns) **b**= $\left[ \begin{matrix} 1 & 0 \\ \begin{matrix} 0 \\ 0 \end{matrix} & \begin{matrix} 1 \\ 1 \end{matrix} \end{matrix} \right]$. The product of **a** and **b** would result in **Q** contributions (Quaas, 1988) that shown in Appendix A Table 1. The **Q*** values are zero for the genotyped animals (Appendix A Table 1) due to J* = 0 for these animals. The **Q^+^** values are the same for the non-genotyped animals in a group as these animals have the same J values that were weighted by **Q** values for genotyped animals in the first (0.375) and second (0.625) group. The **Q-Q^+^** values are positive which are as expected since it reflects the fraction of the genetic group contribution that can be predicted from genotype; however, negatives were observed in real pedigree.

**Appendix A Table 1**. Values for the **J** factor, genetic groups and group variants calculated based on the simple pedigree given above

| ID | J | Q | |  | Q* | |  | Q^+^ | |  | Q-Q^+^ | |
| --- | --- | --- | --- | --- | --- | --- | --- | --- | --- | --- | --- | --- |
|  |  | g1 | g2 |  | g1 | g2 |  | g1 | g2 |  | g1 | g2 |
| 1 | 0.6 | 0.5 | 0.5 |  | 0.2 | 0.2 |  | 0.225 | 0.375 |  | 0.275 | 0.125 |
| 2 | 0.6 | 0.25 | 0.75 |  | 0.1 | 0.3 |  | 0.225 | 0.375 |  | 0.025 | 0.375 |
| 3 | 1.0 | 0.375 | 0.625 |  | 0.0 | 0.0 |  | 0.375 | 0.625 |  | 0.0 | 0.0 |

**APPENDIX B**

***Derivation of the Q-Q^+^ correction***

The following derivation follows the derivation of the **J**-factor of Hsu et al. (2017) but introduces genetic groups in their derivation. The results are thus only relevant for situations where both genetic group and **J**-factor corrections are required, which is the case in many situations.

**The matrix of expected genotypes (used to correct the genotype matrix):**

Hsu et al. (2017) correct the centered genotype matrix **M_2_** for a matrix of expected genotypes **1k’**, where **k** is a vector of mean genotypes in the base population. The **k** is generally unknown but is implicitly estimated. In the presence of genetic groups, the matrix of expected genotypes is **QK’.**

Where **Q** is a matrix of group contributions of the animals, and **K** is a matrix of mean genotypes in the base population for each of the markers and each of the groups (a column of mean marker genotypes per group). Note that for the genotyped animals, the matrix of expected genotypes is **Q_2_K’,**

where **Q_2_** is the block of the group contribution matrix **Q** that belongs to genotyped animals. The genotype matrix corrected for base population frequencies within each of the genetic groups is thus **(M_2_ - Q_2_K’).**

**Genetic value of genotyped animals:**

Following Hsu et al. (2017), the additive genetic value of genotyped animals is:

$\boldsymbol{g}_{\boldsymbol{2}}\boldsymbol{=1}\boldsymbol{\beta+}\boldsymbol{M}_{\boldsymbol{2}}\boldsymbol{\alpha}$**,**

where $\beta$ is the genetic value of an animal with all genotypes equal to 0, i.e., equal to the mean of the marker genotypes, and thus expresses the mean of the base population based on the genotypes in **M_2_**. The latter we want to correct towards the mean of the pedigree base population. To this end, we subtract and add the expected genotype matrix multiplied with the SNP effects $\alpha$:

$\mathbf{g}_{\mathbf{2}}\boldsymbol{=1\beta+}\mathbf{Q}_{\mathbf{2}}\mathbf{K}^{\mathbf{'}}\boldsymbol{\alpha+}\mathbf{(M}_{\mathbf{2}}\mathbf{-}\mathbf{Q}_{\mathbf{2}}\boldsymbol{K')\alpha}$**.**

Here, $1\beta+Q_{2}K^{'}\alpha$ is the expected genetic value based on the genetic group contributions (for an animal that belongs for 100% to group j, it would depict the genetic mean of group j).

**Expected genotype matrix of non-genotyped animals**:

Conditional on the matrix of genotype means of the groups **K**, but unconditional on actual genotypes, the expected genotypes of non-genotyped animals is: **Q_1_K’,**

where subscript 1 denotes non-genotyped.

When also including the information on the actual genotypes of genotyped relatives, the expected matrix of genotypes becomes:

$\mathbf{M}_{\mathbf{1}}\mathbf{=}\mathbf{Q}_{\mathbf{1}}\mathbf{K}^{\mathbf{'}}\mathbf{+}\mathbf{A}_{\mathbf{12}}\mathbf{A}_{\mathbf{22}}^{\mathbf{-1}}\mathbf{(}\mathbf{M}_{\mathbf{2}}\mathbf{-}\mathbf{Q}_{\mathbf{2}}\mathbf{K}^{\mathbf{'}}\mathbf{)}$**.**

**Genetic value of non-genotyped animals**

The additive genetic value of non-genotyped animals is:

$\mathbf{g}_{\mathbf{1}}\boldsymbol{=1\beta+}\mathbf{M}_{\mathbf{1}}\boldsymbol{\alpha}\boldsymbol{+\epsilon}$**,**

where $\epsilon$ denotes a residual due to the imperfect imputation of the genotype matrix **M_1_**.

Inserting the above equation for **M_1_** we obtain:

$$\mathbf{g}_{\mathbf{1}}\boldsymbol{=1\beta+}\mathbf{Q}_{\mathbf{1}}\mathbf{K}^{\mathbf{'}}\boldsymbol{\alpha+}\mathbf{A}_{\mathbf{12}}\mathbf{A}_{\mathbf{22}}^{\mathbf{-1}}\mathbf{(}\mathbf{M}_{\mathbf{2}}\mathbf{-}\mathbf{Q}_{\mathbf{2}}\mathbf{K}^{\mathbf{'}}\boldsymbol{)\alpha}\boldsymbol{+\epsilon}$$

**Combining genotyped and non-genotyped animals:**

In matrix form, the combination of genotyped and non-genotyped animals is:

$$\left[ \begin{matrix} \mathbf{g}_{\mathbf{1}} \\ \mathbf{g}_{\mathbf{2}} \end{matrix} \right]\boldsymbol{=1\beta+}\left[ \begin{matrix} \mathbf{Q}_{\mathbf{1}}\mathbf{-}\mathbf{A}_{\mathbf{12}}\mathbf{A}_{\mathbf{22}}^{\mathbf{-1}}\mathbf{Q}_{\mathbf{2}} \\ \mathbf{Q}_{\mathbf{2}}\mathbf{-}\mathbf{Q}_{\mathbf{2}} \end{matrix} \right]\mathbf{K}^{\mathbf{'}}\boldsymbol{\alpha+}\left[ \begin{matrix} \mathbf{A}_{\mathbf{12}}\mathbf{A}_{\mathbf{22}}^{\mathbf{-1}} \\ \mathbf{I} \end{matrix} \right]\mathbf{M}_{\mathbf{2}}\boldsymbol{\alpha+}\left[ \begin{matrix} \boldsymbol{\epsilon} \\ \mathbf{0} \end{matrix} \right]$$

When summarizing the vector $\boldsymbol{K'\alpha}$ into a vector of genetic group effects $\boldsymbol{\mu}_{\boldsymbol{g}}\boldsymbol{=K'\alpha}$, the additive genetic values of the genotyped and non-genotyped animals become:

$\left[ \begin{matrix} \mathbf{g}_{\mathbf{1}} \\ \mathbf{g}_{\mathbf{2}} \end{matrix} \right]\boldsymbol{=1\beta+}\left[ \begin{matrix} \mathbf{Q}_{\mathbf{1}}\mathbf{-}\mathbf{Q}_{\mathbf{1}}^{\mathbf{+}} \\ \mathbf{0} \end{matrix} \right]\boldsymbol{\mu}_{\mathbf{g}}\mathbf{+}\left[ \begin{matrix} \mathbf{A}_{\mathbf{12}}\mathbf{A}_{\mathbf{22}}^{\mathbf{-1}} \\ \mathbf{I} \end{matrix} \right]\mathbf{M}_{\mathbf{2}}\boldsymbol{\alpha+}\left[ \begin{matrix} \boldsymbol{\epsilon} \\ \mathbf{0} \end{matrix} \right]$**,** where $\mathbf{Q}_{\mathbf{1}}^{\mathbf{+}}\mathbf{=}\mathbf{A}_{\mathbf{12}}\mathbf{A}_{\mathbf{22}}^{\mathbf{-1}}\mathbf{Q}_{\mathbf{2}}\mathbf{.}$

This clearly shows the coefficient matrix for the estimation of the genetic group effects $\mu_{g}$. It may also be noted that the base population against which genetic values are expressed here is defined by the centralization of the marker data (ignoring the overall mean $\beta$, an animal with all genotypes equal to 0 obtains a breeding value of 0).

**APPENDIX C**

***The*** $\boldsymbol{Cov}\left( {\hat{\boldsymbol{u}}}_{\boldsymbol{w}}\boldsymbol{,}{\hat{\boldsymbol{u}}}_{\boldsymbol{p}} \right)\boldsymbol{=Var(}{\hat{\boldsymbol{u}}}_{\boldsymbol{p}}\boldsymbol{)}$ ***assumption of the LR method****.*

The LR method relies on the assumption that the variance of the GEBV based on the partial data set, $Var({\hat{\mathbf{u}}}_{\mathbf{p}})$, is expected to equal the covariance between the GEBV from the whole and the partial data set, ${Cov(\hat{\mathbf{u}}}_{w},{\hat{\mathbf{u}}}_{\mathbf{p}})$ (Legarra and Reverter, 2018). Reverter et al. (1994) proved that this assumption holds for BLUP-EBV when the partial data set contains a subset of the phenotypic records of the whole data set, i.e., when moving from the partial to the whole data set additional phenotypes become available.

Legarra and Reverter (2018) suggest that the LR method can also be applied when the additional information consists of genotypes instead of phenotypes, but do not prove that the $\mathrm{Cov}\left( {\hat{\mathbf{u}}}_{\mathbf{w}}\mathbf{,}{\hat{\mathbf{u}}}_{\mathbf{p}} \right)\mathbf{=}Var({\hat{\mathbf{u}}}_{\mathbf{p}})$ assumption holds in this case. In this Appendix, we prove that $\mathrm{Cov}\left( {\hat{\mathbf{u}}}_{\mathbf{w}}\mathbf{,}{\hat{\mathbf{u}}}_{\mathbf{p}} \right)=Var({\hat{\mathbf{u}}}_{\mathbf{p}})$ holds in general when breeding value estimates are based on BLUP.

Let ${\hat{\mathbf{u}}}_{\mathbf{p}}$ denote the SSGBLUP breeding value estimate based on the single-step relationship matrix $\mathbf{H}_{\mathbf{p}}$. When moving from the partial to the whole data set additional genotypes become available and the single step relationship matrix and breeding value estimates change into:

$\mathbf{H}_{\mathbf{w}}\boldsymbol{=}\mathbf{H}_{\mathbf{p}}\mathbf{+D}$ [C1]

and

${\hat{\mathbf{u}}}_{\mathbf{w}}\boldsymbol{=}{\hat{\mathbf{u}}}_{\mathbf{p}}\boldsymbol{+}\boldsymbol{\delta}$, [C2]

respectively, where **D** denotes the changes of the relationship matrix **H_p_** due to the extra information and $\boldsymbol{\delta}$ the accompanying changes in breeding value estimates. We decompose the true breeding value, **u**, into its SSGBLUP estimates, ${\hat{\mathbf{u}}}_{\mathbf{p}}$ and ${\hat{\mathbf{u}}}_{\mathbf{w}}$, and a prediction error **E_p_** and **E_w_**, respectively:

$\mathbf{u}={\hat{\mathbf{u}}}_{\mathbf{p}}+\mathbf{E}_{\mathbf{p}}\boldsymbol{=}{\hat{\mathbf{u}}}_{\mathbf{p}}+\boldsymbol{\delta}+\mathbf{E}_{\mathbf{w}}\boldsymbol{=}{\hat{\mathbf{u}}}_{\mathbf{w}}+\mathbf{E}_{\mathbf{w}}$ [C3]

where $\mathbf{E}_{\mathbf{p}}\boldsymbol{=}\mathbf{E}_{\mathbf{w}}\boldsymbol{+\delta}$**.** In the case of BLUP, there is no covariance between the prediction errors and the breeding value estimates (Henderson, 1984): $\mathrm{Cov}\left( {\hat{\mathbf{u}}}_{\mathbf{p}}\mathbf{,}\mathbf{E}_{\mathbf{p}} \right)=Cov\left( {\hat{\mathbf{u}}}_{\mathbf{w}}\mathbf{,}\mathbf{E}_{\mathbf{w}} \right)=0,$ i.e. prediction errors are orthogonal to the breeding value estimates and the information for the prediction of the breeding values is optimally used (denoted by ‘Best’ in the BLUP acronym). In the following, we proof that ${\hat{\mathbf{u}}}_{\mathbf{p}}$ and $\boldsymbol{\delta}$ are independent, i.e.:

$\mathrm{Cov}\left( {\hat{\mathbf{u}}}_{\mathbf{p}}\mathbf{,}\boldsymbol{\delta} \right)=0$. [C4]

*Proof* $\mathrm{Cov}\left( {\hat{\mathbf{u}}}_{\mathbf{p}}\boldsymbol{, \delta} \right)=0$: BLUP breeding values have the property (Henderson, 1984):

$${\hat{\mathbf{u}}}_{\mathbf{p}}=E(\mathbf{u}|\mathbf{y,}\mathbf{H}_{\mathbf{p}})$$

$${\hat{\mathbf{u}}}_{\mathbf{w}}=E(\mathbf{u}|\mathbf{y,}\mathbf{H}_{\mathbf{p}},\mathbf{D})$$

where **y** are the phenotypic records. From conditional distribution theory:

$E\left( \mathbf{u} | \mathbf{y,}\mathbf{H}_{\mathbf{p}} \right)=E_{\mathbf{D|y,}\mathbf{H}_{\mathbf{p}}}[E\left( \mathbf{u} | \mathbf{y,}\mathbf{H}_{\mathbf{p}},\mathbf{D} \right)]$

${\hat{\mathbf{u}}}_{\mathbf{p}}\mathbf{=}E_{\mathbf{D|y,}\mathbf{H}_{\mathbf{p}}}\mathbf{[}{\hat{\mathbf{u}}}_{\mathbf{w}}\mathbf{|y,}\mathbf{H}_{\mathbf{p}}\mathbf{]}$ [C5]

where $E_{\mathbf{D|y,}\mathbf{H}_{\mathbf{p}}}\mathbf{[]}$ denotes expectation over all possible values of **D** given **y** and **H_p_**. Next, we set up a model for ${\hat{\mathbf{u}}}_{\mathbf{w}}$ that equates ${\hat{\mathbf{u}}}_{\mathbf{w}}$ to its expectation plus a residual $\boldsymbol{\epsilon}$:

${\hat{\mathbf{u}}}_{\mathbf{w}}\boldsymbol{=}E_{\mathbf{D|y,}\mathbf{H}_{\mathbf{p}}}\left[ {\hat{\mathbf{u}}}_{\mathbf{w}}\mathbf{|y,}\mathbf{H}_{\mathbf{p}}\mathbf{,D} \right]\boldsymbol{+\epsilon}$ [C6]

${\hat{\mathbf{u}}}_{\mathbf{w}}\boldsymbol{=}{\hat{\mathbf{u}}}_{\mathbf{p}}\boldsymbol{+\epsilon}$

where the latter equality is from Equation [C5]. Note that this residual was already defined as $\boldsymbol{\delta}$ in Equation [C2], and we will continue calling it $\boldsymbol{\delta}$ in the following. From [C6], the residual $\boldsymbol{\delta=\epsilon}$ has expectation **0**, i.e.:

${\mathbf{E}(\boldsymbol{\delta}\mathbf{|y,H}}_{\mathbf{p}}\mathbf{)=0}$

i.e., $\mathbf{y}$ and $\mathbf{H}_{\mathbf{p}}$ cannot predict $\boldsymbol{\delta}$. Since ${\hat{\mathbf{u}}}_{\mathbf{p}}$ is a function of (is calculated from) **y** and **H_p_**, this also implies:

$\boldsymbol{E(\delta|}{\hat{\mathbf{u}}}_{\mathbf{p}}\mathbf{=0)}$ [C7]

Now, applying the conditional covariance formula to $\mathrm{Cov}\left( {\hat{\mathbf{u}}}_{\mathbf{p}}\mathbf{,}\boldsymbol{\delta} \right)$ (conditioning on ${\hat{\mathbf{u}}}_{\mathbf{p}}$) yields:

$\mathrm{Cov}\left( {\hat{\mathbf{u}}}_{\mathbf{p}}\boldsymbol{,\delta} \right)=E_{{\hat{\mathbf{u}}}_{\mathbf{p}}}\left[ \mathrm{Cov}\left( {\hat{\mathbf{u}}}_{\mathbf{p}}\boldsymbol{,\delta|}{\hat{\mathbf{u}}}_{\mathbf{p}} \right) \right]+Cov_{{\hat{\mathbf{u}}}_{\mathbf{p}}}[E\left( {\hat{\mathbf{u}}}_{\mathbf{p}} | {\hat{\mathbf{u}}}_{\mathbf{p}} \right),E\left( \boldsymbol{\delta} | {\hat{\mathbf{u}}}_{\mathbf{p}} \right)]$ [C8]

where the second term equals 0 because of [C7]. The $\mathrm{Cov}\left( {\hat{\mathbf{u}}}_{\mathbf{p}}\mathbf{,}\boldsymbol{\delta|}{\hat{\mathbf{u}}}_{\mathbf{p}} \right)$ term equals:

$$\mathrm{Cov}\left( {\hat{\mathbf{u}}}_{\mathbf{p}}\boldsymbol{,\delta|}{\hat{\mathbf{u}}}_{\mathbf{p}} \right)=E\left( {\hat{\mathbf{u}}}_{\mathbf{p}}\boldsymbol{*\delta} | {\hat{\mathbf{u}}}_{\mathbf{p}} \right)\mathbf{-}E\left( {\hat{\mathbf{u}}}_{\mathbf{p}} | {\hat{\mathbf{u}}}_{\mathbf{p}} \right)*E\left( \boldsymbol{\delta} | {\hat{\mathbf{u}}}_{\mathbf{p}} \right)$$

$$\mathrm{Cov}\left( {\hat{\mathbf{u}}}_{\mathbf{p}}\boldsymbol{,\delta|}{\hat{\mathbf{u}}}_{\mathbf{p}} \right)=E\left( {\hat{\mathbf{u}}}_{\mathbf{p}}\boldsymbol{*\delta} | {\hat{\mathbf{u}}}_{\mathbf{p}} \right)\mathbf{=}{\hat{\mathbf{u}}}_{\mathbf{p}}*E\left( \boldsymbol{\delta} | {\hat{\mathbf{u}}}_{\mathbf{p}} \right)\mathbf{=0}$$

due to repeatedly applying [C7]. Hence, all terms in the right-hand-side of [C8] are 0, and we thus have:

$$\mathrm{Cov}\left( {\hat{\mathbf{u}}}_{\mathbf{p}}\boldsymbol{,\delta} \right)=\mathbf{0}$$

*Q.E.D.*

Equation [C4] (and [C7]) implies that we cannot predict (from ${\hat{\mathbf{u}}}_{\mathbf{p}}$**)** the change in breeding values that comes when moving from partial to whole data. In the case of additional genotypes, this implies that prior to the extra genotypes our expectation of the relationship matrix is **H_p_** and afterwards the genotypes become available it is **H_w_**, i.e., E(**H_w_**)= **H_p_**. The change in relationship estimates **D**=**H_w_**-**H_p_** is on average (given initial estimates **H_p_**): E(**H_w_**)- **H_p_** = **0** and cannot be predicted from the initial relationships **H_p_** (otherwise an improved initial relationship matrix could have been constructed). In case, the partial data set contains no genotypes and the whole data set all, **H_p_=A** and **H_w_=G**, we have the well-known result E(**G**)=**A** (Powell et al., 2010). Also, if the partial data set has missing pedigree information that becomes available in the whole data set, the methods to construct relationship matrices with unknown parent groups (**A_UPG_**) attempt to predict the relationships between the animals such that on average **A_UPG_** equals **A** (VanRaden, 1992), i.e., E(**A**)= **A_UPG_**. For instance, an unknown parent from group *i* obtains an inbreeding coefficient, *F_i_*, i.e., the average inbreeding in group *i*. If the missing parent becomes known, it may have a higher or a lower inbreeding than *F_i_*, but on average its inbreeding will equal *F_i_*.

**APPENDIX D**

***QP transformation of Q – Q^+^ group contributions yields Altered-QP H inverse of Masuda et al. (2021)***

The **“**Q – Q^+^**”** genetic group regression coefficients are:

$$\mathbf{Q}=\mathbf{Q -}\mathbf{Q}^{\mathbf{+}} =\left[ \begin{matrix} \mathbf{Q}_{\mathbf{1}}\mathbf{-}\mathbf{Q}_{\mathbf{1}}^{\mathbf{+}} \\ \mathbf{Q}_{\mathbf{2}}\mathbf{-}\mathbf{Q}_{\mathbf{2}}^{\mathbf{+}} \end{matrix} \right]=\left[ \begin{matrix} \mathbf{Q}_{\mathbf{1}}\mathbf{-}\mathbf{A}_{\mathbf{12}}\mathbf{A}_{\mathbf{22}}^{\mathbf{-1}}\mathbf{Q}_{\mathbf{2}} \\ \mathbf{0} \end{matrix} \right]$$

Where 1 and 2 denote non-genotyped and genotyped respectively, and **Q_1_** (**Q_2_**) is the traditional genetic group coefficient matrix for the non-genotyped (genotyped) animals.

The $\mathbf{H}^{\mathbf{-1}}$ matrix after QP transformation of genetic group effects is:

$$\mathbf{H}^{\mathbf{-1}}\mathbf{=}\left[ \begin{matrix} \mathbf{A}^{\mathbf{11}} & \mathbf{A}^{\mathbf{12}} & \mathbf{-}\mathbf{A}^{\mathbf{11}}\mathbf{Q}_{\mathbf{1}}\mathbf{-}\mathbf{A}^{\mathbf{12}}\mathbf{Q}_{\mathbf{2}} \\ \mathbf{A}^{\mathbf{21}} & \mathbf{A}^{\mathbf{22}}\mathbf{+}\mathbf{G}^{\mathbf{-1}}\mathbf{-}\mathbf{A}_{\mathbf{22}}^{\mathbf{-1}} & \mathbf{-}\mathbf{A}^{\mathbf{21}}\mathbf{Q}_{\mathbf{1}}\mathbf{-}\mathbf{A}^{\mathbf{21}}{\mathbf{A}^{\mathbf{11}}}^{\mathbf{-1}}\mathbf{A}^{\mathbf{12}}\mathbf{Q}_{\mathbf{2}} \\ \mathbf{-}{\mathbf{Q'}_{\mathbf{1}}\mathbf{A}}^{\mathbf{11}}\mathbf{-}{\mathbf{Q'}_{\mathbf{2}}\mathbf{A}}^{\mathbf{21}} & \mathbf{-}{\mathbf{Q'}_{\mathbf{1}}\mathbf{A}}^{\mathbf{12}}\mathbf{-}{\mathbf{Q'}_{\mathbf{2}}\mathbf{A}}^{\mathbf{12}}{\mathbf{A}^{\mathbf{11}}}^{\mathbf{-1}}\mathbf{A}^{\mathbf{21}} & \mathbf{Q}_{\mathbf{1}}^{\mathbf{'}}\mathbf{A}^{\mathbf{11}}\mathbf{Q}_{\mathbf{1}}\mathbf{+}\mathbf{Q}_{\mathbf{2}}^{\mathbf{'}}\mathbf{A}^{\mathbf{21}}{\mathbf{A}^{\mathbf{11}}}^{\mathbf{-1}}\mathbf{A}^{\mathbf{12}}\mathbf{Q}_{\mathbf{2}}\mathbf{+}\mathbf{Q}_{\mathbf{2}}^{\mathbf{'}}\mathbf{A}^{\mathbf{21}}\mathbf{Q}_{\mathbf{1}}\mathbf{+}\mathbf{Q}_{\mathbf{1}}^{\mathbf{'}}\mathbf{A}^{\mathbf{12}}\mathbf{Q}_{\mathbf{2}} \end{matrix} \right]$$

Note that $\mathbf{A}_{\mathbf{22}}^{\mathbf{-1}}\mathbf{=}\mathbf{A}^{\mathbf{22}}\mathbf{-A}^{\mathbf{21}}{\mathbf{A}^{\mathbf{11}}}^{\mathbf{-1}}\mathbf{A}^{\mathbf{12}}$**, i.e.,** $\mathbf{A}^{\mathbf{21}}{\mathbf{A}^{\mathbf{11}}}^{\mathbf{-1}}\mathbf{A}^{\mathbf{12}}\mathbf{=}\mathbf{A}^{\mathbf{22}}\mathbf{-}\mathbf{A}_{\mathbf{22}}^{\mathbf{-1}}$. Substituting the last expression to the lower right-hand corner gives

$\mathbf{Q}_{\mathbf{1}}^{\mathbf{'}}\mathbf{A}^{\mathbf{11}}\mathbf{Q}_{\mathbf{1}}\mathbf{+}\mathbf{Q}_{\mathbf{2}}^{\mathbf{'}}\mathbf{A}^{\mathbf{21}}\mathbf{Q}_{\mathbf{1}}\mathbf{+}\mathbf{Q}_{\mathbf{1}}^{\mathbf{'}}\mathbf{A}^{\mathbf{12}}\mathbf{Q}_{\mathbf{2}}\mathbf{+}\mathbf{Q}_{\mathbf{2}}^{\mathbf{'}}\mathbf{A}^{\mathbf{22}}\mathbf{Q}_{\mathbf{2}}\mathbf{-}\mathbf{Q}_{\mathbf{2}}^{\mathbf{'}}\mathbf{A}_{\mathbf{22}}^{\mathbf{-1}}\mathbf{Q}_{\mathbf{2}}\mathbf{=}\mathbf{Q}^{\mathbf{'}}\mathbf{A}^{\mathbf{-1}}\mathbf{Q}\mathbf{-}\mathbf{Q}_{\mathbf{2}}^{\mathbf{'}}\mathbf{A}_{\mathbf{22}}^{\mathbf{-1}}\mathbf{Q}_{\mathbf{2}}$**.**

The (2,3)-block of $\mathbf{H}^{\mathbf{-1}}$ can be written as $\mathbf{-}\mathbf{A}^{\mathbf{21}}\mathbf{Q}_{\mathbf{1}}\mathbf{-}\mathbf{A}^{\mathbf{22}}\mathbf{Q}_{\mathbf{2}}\mathbf{+}\mathbf{A}_{\mathbf{22}}^{\mathbf{-1}}\mathbf{Q}_{\mathbf{2}}$**.** Taking these results, we can write as

$\mathbf{H}^{\mathbf{-1}}\mathbf{=}\left[ \begin{matrix} \mathbf{A}^{\mathbf{11}} & \mathbf{A}^{\mathbf{12}} & \mathbf{-}\mathbf{A}^{\mathbf{11}}\mathbf{Q}_{\mathbf{1}}\mathbf{-}\mathbf{A}^{\mathbf{12}}\mathbf{Q}_{\mathbf{2}} \\ \mathbf{A}^{\mathbf{21}} & \mathbf{A}^{\mathbf{22}}\mathbf{+}\mathbf{G}^{\mathbf{-1}}\mathbf{-}\mathbf{A}_{\mathbf{22}}^{\mathbf{-1}} & \mathbf{-}\mathbf{A}^{\mathbf{21}}\mathbf{Q}_{\mathbf{1}}\mathbf{-}\mathbf{A}^{\mathbf{22}}\mathbf{Q}_{\mathbf{2}}\mathbf{+}\mathbf{A}_{\mathbf{22}}^{\mathbf{-1}}\mathbf{Q}_{\mathbf{2}} \\ \mathbf{-}{\mathbf{Q'}_{\mathbf{1}}\mathbf{A}}^{\mathbf{11}}\mathbf{-}{\mathbf{Q'}_{\mathbf{2}}\mathbf{A}}^{\mathbf{21}} & \mathbf{-}\mathbf{Q'}_{\mathbf{1}}\mathbf{A}^{\mathbf{12}}\mathbf{-}{\mathbf{Q'}_{\mathbf{2}}\mathbf{A}}^{\mathbf{22}}\mathbf{+}\mathbf{Q'}_{\mathbf{2}}\mathbf{A}_{\mathbf{22}}^{\mathbf{-1}} & \mathbf{Q}^{\mathbf{'}}\mathbf{A}^{\mathbf{-1}}\mathbf{Q}\mathbf{-}\mathbf{Q}_{\mathbf{2}}^{\mathbf{'}}\mathbf{A}_{\mathbf{22}}^{\mathbf{-1}}\mathbf{Q}_{\mathbf{2}} \end{matrix} \right]$ or

$\mathbf{H}^{\mathbf{-1}}\mathbf{=}\left[ \begin{matrix} \mathbf{A}^{\mathbf{-1}} & \mathbf{-}\mathbf{A}^{\mathbf{-1}}\mathbf{Q} \\ \mathbf{-}\mathbf{Q'A}^{\mathbf{-1}} & \mathbf{Q'}\mathbf{A}^{\mathbf{-1}}\mathbf{Q} \end{matrix} \right]\mathbf{+}\left[ \begin{matrix} \boldsymbol{0} & \boldsymbol{0} & \boldsymbol{0} \\ \boldsymbol{0} & \mathbf{G}^{\mathbf{-1}} & \boldsymbol{0} \\ \boldsymbol{0} & \boldsymbol{0} & \boldsymbol{0} \end{matrix} \right]\mathbf{-}\left[ \begin{matrix} \boldsymbol{0} & \boldsymbol{0} & \boldsymbol{0} \\ \boldsymbol{0} & \mathbf{A}_{\mathbf{22}}^{\mathbf{-1}} & \mathbf{-A}_{\mathbf{22}}^{\mathbf{-1}}\mathbf{Q}_{\mathbf{2}} \\ \boldsymbol{0} & {\mathbf{-}\mathbf{Q'}_{\mathbf{2}}\mathbf{A}}_{\mathbf{22}}^{\mathbf{-1}} & \mathbf{Q}_{\mathbf{2}}^{\mathbf{'}}\mathbf{A}_{\mathbf{22}}^{\mathbf{-1}}\mathbf{Q}_{\mathbf{2}} \end{matrix} \right]$,

which is the same as the Altered QP H inverse of Masuda et al. (2021), except that Masuda et al. used Vitezica et al. (2011)’s scaled **G** matrix which implies a random correction for the **J** factor. It is not clear from their paper why this additional correction for the **J** factor, on top of that implied by the (**Q-Q^+^**) matrix (see Appendix B), would be needed.

***QP transformation of Q^*^ group contributions***

The Q* genetic group contributions are defined in the main text and are:

$$\mathbf{Q}^{\mathbf{*}}\mathbf{=}\left[ \begin{aligned} \mathbf{Q}_{\mathbf{1}}^{\mathbf{*}} \\ \boldsymbol{0} \end{aligned} \right]$$

The $\mathbf{H}^{\mathbf{-1}}$ matrix after QP transformation of **Q*** effects is:

$\mathbf{H}^{\mathbf{-1}}\mathbf{=}\left[ \begin{matrix} \mathbf{A}^{\mathbf{11}} & \mathbf{A}^{\mathbf{12}} & \mathbf{-A}^{\mathbf{11}}\mathbf{Q}_{\mathbf{1}}^{\mathbf{*}} \\ \mathbf{A}^{\mathbf{21}} & \mathbf{A}^{\mathbf{22}}\mathbf{+}\mathbf{G}^{\mathbf{-1}}\mathbf{-}\mathbf{A}_{\mathbf{22}}^{\mathbf{-1}} & \mathbf{-A}^{\mathbf{21}}\mathbf{Q}_{\mathbf{1}}^{\mathbf{*}} \\ {\mathbf{-Q}_{\mathbf{1}}^{\mathbf{*}}}^{\mathbf{'}}\mathbf{A}^{\mathbf{11}} & {\mathbf{-Q}_{\mathbf{1}}^{\mathbf{*}}}^{\mathbf{'}}\mathbf{A}^{\mathbf{12}} & {\mathbf{Q}_{\mathbf{1}}^{\mathbf{*}}}^{\mathbf{'}}\mathbf{A}^{\mathbf{11}}\mathbf{Q}_{\mathbf{1}}^{\mathbf{*}} \end{matrix} \right]$**.**

Where 1 and 2 denote non-genotyped and genotyped, respectively.

**References only cited in Appendices:**

Henderson, C. 1984. Application of linear models in animal breeding. University of Guelph.

VanRaden, P. J. J. o. D. S. 1992. Accounting for inbreeding and crossbreeding in genetic evaluation of large populations. J Dairy Sci 75(11):3136-3144. <https://doi.org/3110.3168/jds.S0022-0302(3192)78077-78071>.
